# Deleterious Role of Th9 Cells in Pulmonary Fibrosis

**DOI:** 10.3390/cells10113209

**Published:** 2021-11-17

**Authors:** Kui Miao Deng, Xiang Sheng Yang, Qun Luo, Yi Xin She, Qing Yang Yu, Xiao Xiao Tang

**Affiliations:** State Key Laboratory of Respiratory Disease, National Clinical Research Center for Respiratory Disease, National Center for Respiratory Medicine, Guangzhou Institute of Respiratory Health, The First Affiliated Hospital of Guangzhou Medical University, Guangzhou 510182, China; dengkuimiao@163.com (K.M.D.); xiangshy2008@126.com (X.S.Y.); luoqunx@163.com (Q.L.); sheyixinsemail@163.com (Y.X.S.); yuqingyang0413@163.com (Q.Y.Y.)

**Keywords:** idiopathic pulmonary fibrosis, Th9 cells, Th2 cells, interleukin-9

## Abstract

Idiopathic pulmonary fibrosis (IPF) is a progressive and fatal lung disease of unknown etiology. Immune disorders play an important role in IPF pathogenesis. Here, we show that Th9 cells differentiate and activate in the lung tissue of patients with IPF and bleomycin (BLM)-induced lung fibrosis mice. Moreover, we found that Th9 cells promote pulmonary fibrosis in two ways. On the one hand, Th9 cells promote fibroblast differentiation, activation, and collagen secretion by secreting IL-9. On the other hand, they promote differentiation of Th0 cells into Th2 cells by secreting IL-4. Th9 cells and Th2 cells can promote each other, accelerating the Th1/Th2 imbalance and eventually forming a positive feedback of pulmonary fibrosis. In addition, we found that neutralizing IL-9 in both preventive and therapeutic settings ameliorates bleomycin-induced pulmonary fibrosis. Furthermore, we identified several critical signaling pathways involved in the effect of neutralizing IL-9 on pulmonary fibrosis by proteomics study. From an immunological perspective, we elucidated the novel role and underlying mechanism of Th9 cells in pulmonary fibrosis. Our study suggested that Th9-based immunotherapy may be employed as a treatment strategy for IPF.

## 1. Introduction

Idiopathic pulmonary fibrosis (IPF) is a chronic, progressive, incurable, and fatal disease characterized by pulmonary fibrosis [1]. Its main pathological feature is persistent alveolar epithelial cell injury, as well as abnormal proliferation and activation of fibroblasts [2]. Excessive extracellular matrix deposition and structural destruction of lung tissue lead to fibrosis and dysfunction of the lung, respiratory failure, and eventually death. The incidence of IPF has continued to increase in recent years and the disease course is irreversible. IPF is more common in middle-aged and older males, with a poor prognosis and a median survival of only 2–3 years [3]. The pathogenesis of IPF is not completely understood, and currently there is no effective treatment. The two oral drugs approved by the US FDA, nintedanib and pirfenidone, can only relieve symptoms or slow disease progression. The latest guidelines just weakly recommended them for IPF drug treatment [1]. In recent years, several drugs have been tested in clinical trials, but their effects are limited [4]. Therefore, development of new and effective treatment strategies has been of great significance and is desperately needed.

Innate and acquired immune responses play an important role in the pathogenesis of IPF [5]. T cells are widely present in active disease areas and tertiary lymphatic structures in patients with IPF [6]. CD4^+^ helper T cells (Th cells) are divided into different subtypes according to the type of cytokines they produce. Stimulated by antigen, resting CD4^+^ T cells (Th0 cells) can further differentiate into Th1 and Th2 cells under the influence of polarization signals in the microenvironment. IFN-γ promotes Th1 differentiation, while IL-4 promotes Th2 differentiation. Th1 and Th2 cells cross-inhibit each other and form a negative feedback loop. Th1 cells and Th2 cells each secrete a variety of cytokines, forming a complex cytokine network to regulate the immune response. The cytokines secreted by Th1 cells mainly include IL-2, IFN-γ, TNF, IL-12, and IL-18, while the cytokines secreted by Th2 cells mainly include IL-4, IL-5, IL-6, IL-10, IL-13, and monocyte chemotactic protein-1 (MCP-1). Th1 and Th2 immune responses exert negative feedback through the secretion of cytokines and maintain the immune balance. In recent years, Th1/Th2 imbalance has been shown to play an important role in the pathogenesis of pulmonary fibrosis. During fibrosis, Th1 cells inhibit the proliferation of fibroblasts and the formation of fibrous tissue by secreting anti-fibrotic factors such as IFN-γ and IL-12 [7,8]. IL-4 secreted by Th2 cells has been shown to induce fibroblast aggregation, and promote fibroblast activation and proliferation. Another Th2 cytokine, IL-13, can induce lung fibrosis by selectively stimulating and activating TGF-β [9,10,11,12]. These studies indicated that the Th1 response is anti-fibrotic, whereas the Th2 response is pro-fibrotic. Several studies have confirmed an increased level of Th2 cytokines in the serum of patients with pulmonary fibrosis as compared to that of health controls [13,14,15], indicating that the progression of pulmonary fibrosis may be related to a strong Th2 pro-fibrotic response [16].

Previous studies have shown that TGF-β and IL-4 levels are elevated in patients with IPF [12,17]. Th9 cells, a new CD4^+^ T cell subset, can either be differentiated directly from resting CD4^+^ (Th0) cells stimulated by IL-4 and TGF-β or can be transdifferentiated from Th2 cells stimulated by TGF-β [18]. Therefore, elevated TGF-β and IL-4 may lead to an increased Th9 differentiation in the lungs of patients with IPF. However, the role of Th9 cells in the pathogenesis of pulmonary fibrosis has not yet been explored and determined. A study showed that Th9 cells have pro-inflammatory and anti-inflammatory effects in autoimmune diseases [19]. The main cytokine secreted by Th9 cells is IL-9 [20], which exerts its effects on a variety of cells, and whether it promotes pulmonary fibrosis is also controversial. On the one hand, IL-9 can recruit mast cells and promote their secretion of TGF-β [20,21]. It can also promote fibrosis by inhibiting the antigen-presenting cell-mediated Th1-type immune response and downregulating the expression of anti-fibrotic molecules such as IL-12 and IFN-γ [22]. On the other hand, IL-9 exerts an anti-fibrotic effect by inducing differentiation of monocyte-macrophage and synthesis of an anti-fibrotic molecule PGE2 [23]. In animal studies, Sugimoto et al. found that IL-9 neutralizing antibody ameliorated silica-induced pulmonary fibrosis in mice [24]. Arras et al. found that IL-9 has a protective role in pulmonary fibrosis by comparing IL-9 overexpressing transgenic mice (Tg5 mice) and wild-type controls [25]. In a clinical study, Li et al. found that the serum level of IL-9 in patients with IPF during infection phase was significantly higher than that in the stable phase and healthy controls, suggesting that IL-9 is a risk factor for patients with IPF [26]. Similarly, Jiang et al. measured serum IL-9 levels in 61 patients with connective tissue disease-associated interstitial lung disease (CTD-ILD) and found that their serum IL-9 levels were negatively correlated with lung function [27]. Unlike the above studies, Koichi et al. measured serum IL-9 levels in 71 patients with systemic sclerosis (SSc) and found that IL-9 is a protective factor for patients with SSc [28]. Therefore, more in-depth research is needed to determine whether IL-9 is pro-fibrotic or anti-fibrotic.

Other than secreting IL-9, one study suggested that Th9 cells also secrete some Th2 type cytokines such as IL-4 [29]. An elevated IL-4 has been reported in bronchoalveolar lavage fluid (BALF) from patients with pulmonary fibrosis [12]. Additionally, anti-IL-4 chimeric protein has an anti-fibrotic effect after nasal drip into BLM mice [30].

Based on the above evidence, we hypothesized that Th9 cells abnormally differentiate and activate when TGF-β is abundantly enriched in pulmonary fibrosis. On the one hand, Th9 cells and Th2 cells promote each other (may via IL-4), accelerating the Th1/Th2 imbalance and forming a positive feedback of the pro-fibrosis process. On the other hand, Th9 cells secrete IL-9, which promotes the proliferation and activation of fibroblasts as well as collagen secretion, eventually leading to pulmonary fibrosis. Our hypothesis is different from regarding Th9/IL-9 as a whole in previous studies, and this study is the first to explore the role of Th9 cells in promoting pulmonary fibrosis via IL-9 and IL-4.

## 2. Results

### 2.1. Th9 Differentiation and Activation Increase in the PBMC and Lung Tissue of Patients with IPF

**Figure 1:**
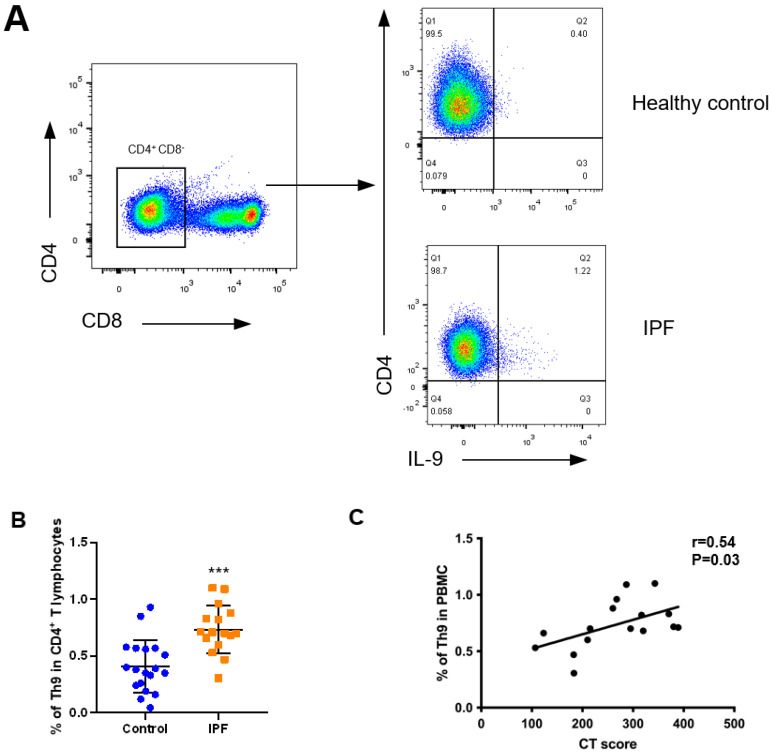

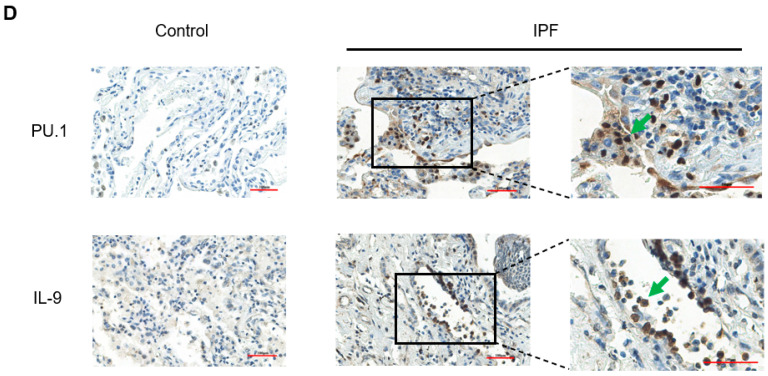
Increased Th9 cell differentiation and IL-9 expression in patients with IPF. (**A**) Representative pseudo-color plot showing proportion of Th9 cells to CD4^+^ T cells in PBMC of patients with IPF and healthy controls by flow cytometry. (**B**) Proportions of Th9 cells to CD4^+^ T cells in PBMC between patients with IPF (*n* = 16) and healthy controls (*n* = 19). (**C**) Correlation analysis between proportion of Th9 cells in PBMC and HRCT score of pulmonary fibrosis (*n* = 16). (**D**) Representative images of PU.1 and IL-9 immunostaining in serial sections of lung tissue from patients with IPF (*n* = 14) and controls (*n* = 4). Scale bars, 100 μm. PBMC, peripheral blood mononuclear cell. HRCT, high resolution CT. *** *p* < 0.001.

Immune disorder has been regarded as one of the important pathogeneses of IPF. Previous studies have shown that TGF-β and IL-4 are elevated in patients with IPF (TGF-β is increased in lung tissue and IL-4 is increased in BALF). The differentiation of Th9 cells depends on TGF-β and IL-4 [12,17], therefore, we speculated that Th9 differentiation increases in the patients of IPF. In order to determine the Th9 ratio in patients with IPF, we isolated peripheral blood mononuclear cells (PBMC) from the whole blood and analyzed it by flow cytometry (Figure 1A) (Table 1). The results showed that the proportion of Th9 cells in CD4^+^ T lymphocytes in the PBMC of patients with IPF was significantly higher than that in healthy controls (Figure 1B) and positively correlated with CT score (Figure 1C), indicating that the increase of Th9 cells is positively correlated with the severity of pulmonary fibrosis in patients with IPF.

To clarify whether the distribution and differentiation of Th9 cells are altered in the lungs of patients with IPF, we examined PU.1 (the specific transcription factor of Th9 cells) in the lung tissues of patients with IPF (*n* = 14) and controls (*n* = 4) by immunohistochemistry (Table 2). As expected, the expression of PU.1 in the lungs of patients with IPF was significantly increased as compared to the controls (Figure 1D), indicating an increased differentiation of Th9 cells in the lungs of patients with IPF. As the main cytokine secreted by Th9 cells is IL-9, we then examined the expression and distribution of IL-9 in the lung tissue of patients with IPF (*n* = 14) and controls (*n* = 4) by immunohistochemistry. The results showed an increased IL-9 expression in the lungs of patients with IPF as well (Figure 1D).

### 2.2. Th9 Differentiation and Activation Increase in Lung Tissue of BLM-Induced Lung Fibrosis Mice

**Figure 2:**
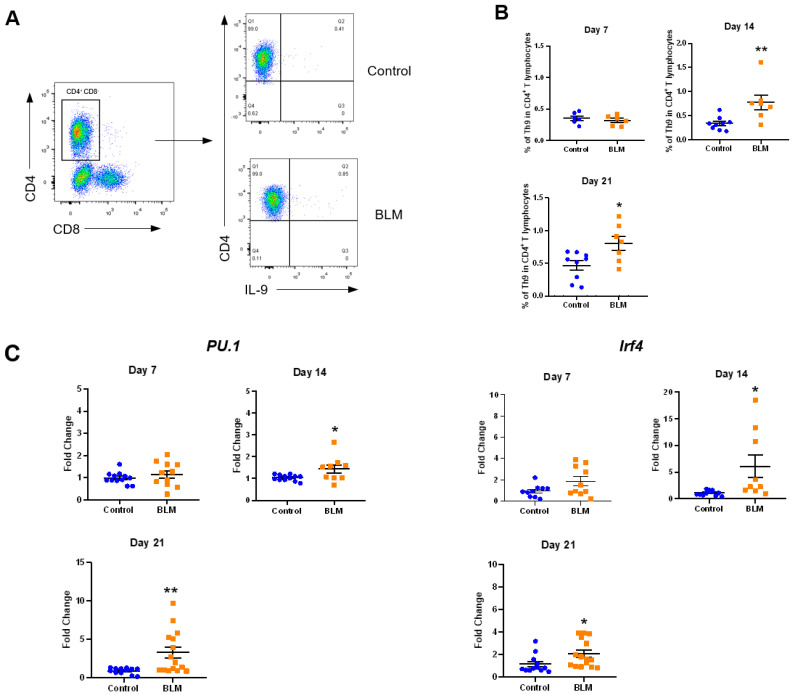

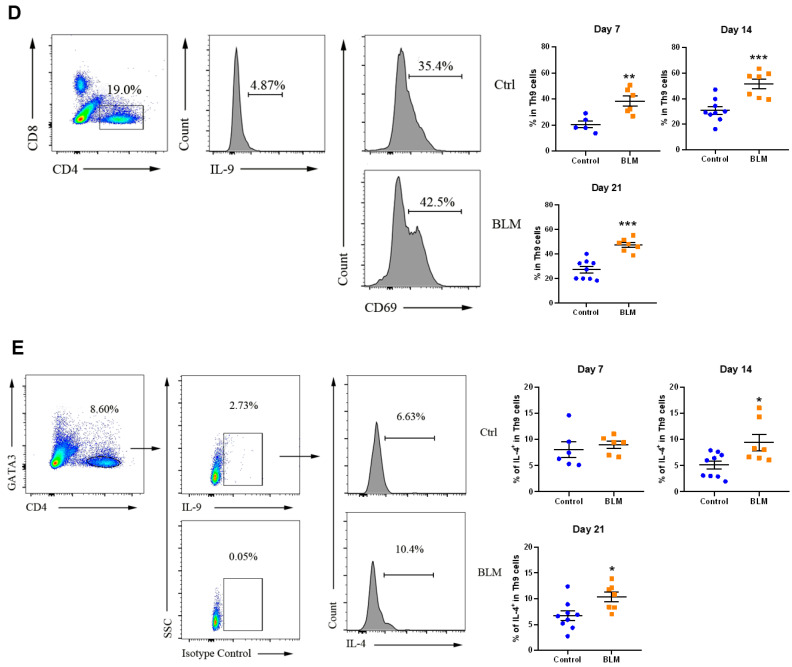
Increased Th9 cell differentiation and activation in the lung of bleomycin (BLM)-induced pulmonary fibrosis mice. (**A**) Representative pseudo-color plot showing proportion of Th9 cells to CD4^+^ T cells in the lungs of BLM mice and control mice (CD4^+^ IL-9^+^ T cells were defined as Th9 cells). (**B**) Proportion of Th9 cells in the lung tissue of BLM mice and control mice (*n* = 6 to 9) at the indicated time points calculated by flow cytometry. (**C**) Quantitative PCR analysis of *PU.1* and *Irf4* in the BALF cells of BLM mice and control mice at the indicated time points. (**D**) Activation of Th9 cells in the lung of BLM mice and control mice at the indicated time points. (**E**) Increased expression of IL-4 in Th9 cells (CD4^+^ IL-9^+^ IL-4^+^ T cells) in the lung of BLM mice. *p* values were determined by two-sided Student’s *t*-test. BALF, bronchoalveolar lavage fluid. * *p* < 0.05, ** *p* < 0.01, *** *p* < 0.001.

We then harvested the lungs of BLM mice on day 7, 14, and 21 after BLM treatment and analyzed the content and activation of Th9 cells by flow cytometry (Figure 2A). We found that starting from day 14 after BLM treatment, the proportion of Th9 cells in CD4^+^ T lymphocytes in the lungs of the model group was significantly higher than that in the control group (control group: 0.3413 ± 0.04497%, model group: 0.7787 ± 0.1534%, *p* < 0.01) and this trend persisted until day 21 after BLM treatment (day 21: control group: 0.4669 ± 0.0714%, model group: 0.8054 ± 0.1093%, *p* < 0.05) (Figure 2B). These results demonstrated that the number of Th9 cells in the lung tissue of BLM mice increased. We also examined the expression level of *PU.1* and *Irf4*, two transcription factors important for Th9 differentiation, in BALF cells of BLM mice and control mice. We found that, starting from day 14 after treatment, the expression levels of *PU.1* and *Irf4* in the BLM-treated group were significantly higher than those in the control group (*PU.1*: day 14, *p* < 0.05; day 21, *p* < 0.01; *Irf4*: day 14, *p* < 0.05; day 21, *p* < 0.05) (Figure 2C). Consistent with this, the flow cytometry results also suggested that Th9 cell activation in the lungs of BLM mice was significantly higher than that in the control group (day 7, *p* < 0.01; day 14, *p* < 0.001; day 21, *p* < 0.0001) (Figure 2D), indicating an increased number and function of Th9 cells in the lungs of BLM mice. In addition, previous studies have shown that various CD4^+^ T cell subsets are not stable and mutual transformation is observed among these subsets [31]. Th9 cell subset can also transform into other Th cell subsets in a polarized microenvironment [32]. Therefore, we tested whether Th9 cells in the lungs of BLM mice functioned as other subsets, especially Th2 subset. In addition to IL-9, we found that Th9 cells in the model group had a higher proportion of CD4^+^IL9^+^IL-4^+^ T cell subset, suggesting an increased IL-4 secretion by Th9 cells in the lungs of BLM mice (day 14: control group: 5.106 ± 0.7764%, model group: 9.391 ± 1.528%, *p* < 0.05; day 21: control group: 6.700 ± 0.9240%, model group: 10.33 ± 0.9359%, *p* < 0.05) (Figure 2E).

### 2.3. IL-9 Promotes Fibroblast Proliferation and Activation In Vitro

**Figure 3:**
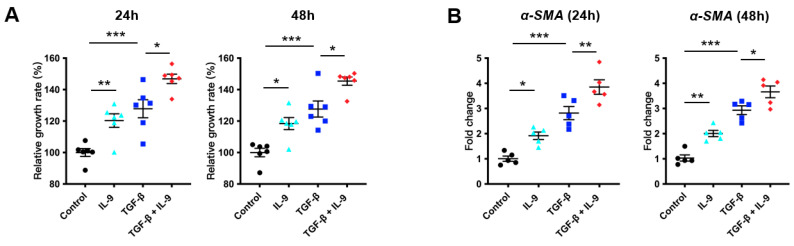

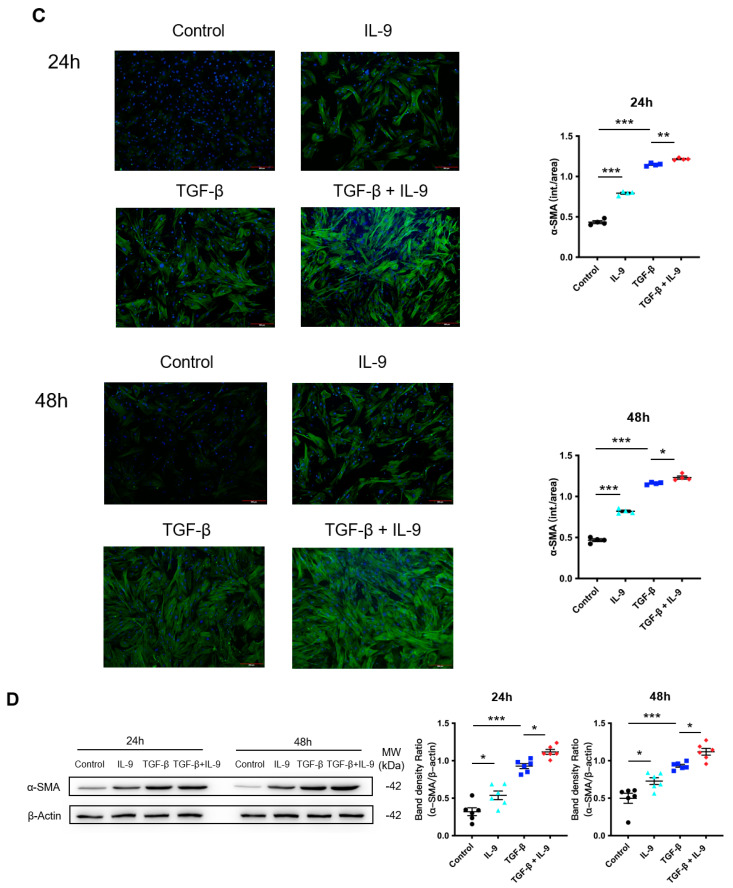

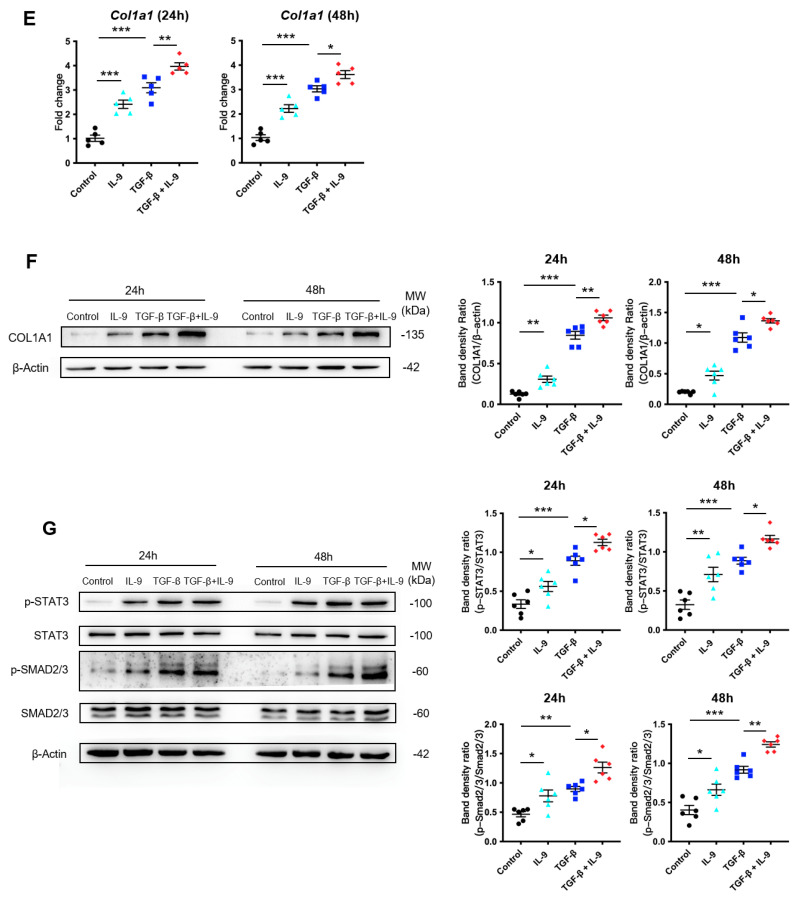
IL-9 promotes fibroblast proliferation and activation in vitro. (**A**) IL-9 (10 ng/mL, 24 h or 48 h) increased proliferation of primary mouse lung fibroblasts in the presence or absence of TGF-β (5 ng/mL) examined by CCK8 assay. (**B**) Quantitative PCR analysis of *α-SMA* in primary mouse lung fibroblasts treated with IL-9 (10 ng/mL, 24 h or 48 h) in the presence or absence of TGF-β (5 ng/mL). (**C**) Representative images and quantitative analysis of α-SMA immunostaining in primary mouse lung fibroblasts treated with IL-9 (10 ng/mL, 24 h or 48 h) in the presence or absence of TGF-β (5 ng/mL) (*n* = 4). Scale bars, 200 μm. (**D**) Representative Western blot images and quantitative analysis of α-SMA in primary mouse lung fibroblasts treated with IL-9 (10 ng/mL, 24 h or 48 h) in the presence or absence of TGF-β (5 ng/mL) (*n* = 6). β-Actin was used as a loading control. (**E**) Quantitative PCR analysis of *Col1a1* in primary mouse lung fibroblasts treated with IL-9 (10 ng/mL, 24 h or 48 h) in the presence or absence of TGF-β (5 ng/mL) (*n* = 5). (**F**) Representative Western blot images and quantitative analysis of COL1A1 in primary mouse lung fibroblasts treated with IL-9 (10 ng/mL, 24 h or 48 h) in the presence or absence of TGF-β (5 ng/mL) (*n* = 6). β-Actin was used as a loading control. (**G**) Representative Western blot images and quantitative analysis of STAT3 and SMAD2/3 phosphorylation and their total expression in primary mouse lung fibroblasts treated with IL-9 (10 ng/mL, 24 h or 48 h) in the presence or absence of TGF-β (5 ng/mL) (*n* = 6). Data were expressed as mean ± SEM. * *p* < 0.05; ** *p* < 0.01, *** *p* < 0.001. *p* values were determined by one-way ANOVA (Tukey’s test).

Previous studies have shown that IL-9 promotes cell proliferation [33,34]. Lung fibroblasts, the major effector cells of the progressive fibrotic process in IPF, secrete excess extracellular matrix, and eventually lead to pulmonary fibrosis. In order to test the effect of IL-9 on the proliferation of fibroblasts, we isolated lung fibroblasts from wild-type mice and stimulated them with IL-9 alone or in the presence of TGF-β (TGF-β was used to mimic the lung microenvironment in pulmonary fibrosis) for 24 or 48 h, and the cell proliferation rate was determined by CCK8 assay. The results showed that 24 h after IL-9 stimulation, the cell proliferation rate was significantly increased as compared to the control group, either in the absence (*p* = 0.0108) or presence (*p* = 0.0144) of TGF-β. 48 h after stimulation, the change trend of each group was consistent with that of 24 h (Figure 3A). These data demonstrated that IL-9 alone or together with TGF-β can promote the proliferation of mouse primary lung fibroblasts in vitro. During the development of pulmonary fibrosis, fibroblasts differentiate into myofibroblasts and secrete more collagen. To clarify whether IL-9 activates fibroblasts, we stimulated fibroblasts with IL-9 alone or in the presence of TGF-β. Real-time quantitative PCR was used to detect the expression of *α-SMA*, which is a marker of fibroblast activation. 24 h after stimulation, IL-9, either alone or together with TGF-β, increased the expression level of *α-SMA* as compared to the control group (*p* < 0.001, *p* = 0.0252). 48 h of stimulation showed the same change trend of each group as that of 24 h (Figure 3B). These results indicated that IL-9, either in the absence or presence of TGF-β, can increase activation of mouse primary lung fibroblasts in vitro. In addition to real-time qPCR, we also detected an enhanced expression level of α-SMA by immunofluorescence (Figure 3C). In order to verify the results at the protein level, we detected the α-SMA expression by Western blot and found that it was markedly increased after IL-9 stimulation than that in the control (Figure 3D), corroborated the immunofluorescence results. During the progress of IPF, (myo)fibroblasts secrete a large amount of extracellular matrix, including collagen I. In order to determine the effect of IL-9 on collagen I secretion by fibroblasts, we stimulated fibroblasts with IL-9 alone or in the presence of TGF-β for 24 h or 48 h and then detected the *Col1a1* expression by real-time qPCR. The results showed that 24 h after stimulation, *Col1a1* expression in IL-9 group was remarkably higher than that in the control group (*p* < 0.001). The mRNA expression of *Col1a1* in the IL-9 and TGF-β-stimulated group was also significantly higher than that in the TGF-β-stimulated group (*p* = 0.0091). 48 h after stimulation, the change trend of each group was consistent with that of 24 h (Figure 3E). We obtained similar results by Western blot (Figure 3F). These data demonstrated that IL-9 alone or together with TGF-β can promote the *Col1a1* secretion of mouse primary lung fibroblasts in vitro. Previous studies have shown that an increased STAT3 and SMAD2/3 phosphorylation participates in IPF progression [35,36]. We therefore wondered if they are involved in the effect of IL-9 on fibroblasts. The results showed that stimulation of fibroblasts with IL-9 alone or together with TGF-β for 24 h increased the phosphorylation of STAT3 (*p* = 0.043, *p* = 0.0341) and SMAD2/3 (*p* = 0.0366, *p* = 0.0126), and stimulation for 48 h showed a similar pattern (Figure 3G). Taken together, these results indicated that IL-9 can promote proliferation, activation, and collagen secretion of fibroblasts. Moreover, an increased phosphorylation of STAT3 and SMAD2/3 may be involved in the effect of IL-9 on fibroblasts.

### 2.4. Th9 Cells Promote Th0 Cells to Differentiate into Th2 Cells and Induce Lung Fibroblasts to Secrete More Collagen

**Figure 4:**
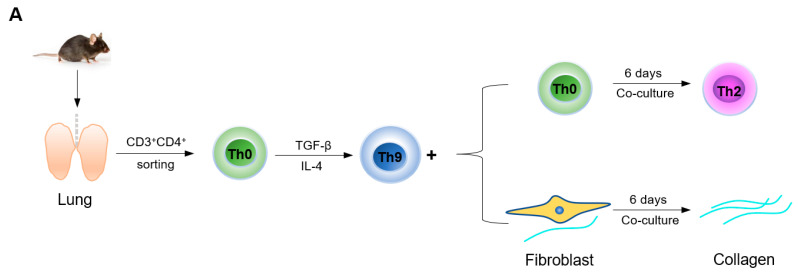

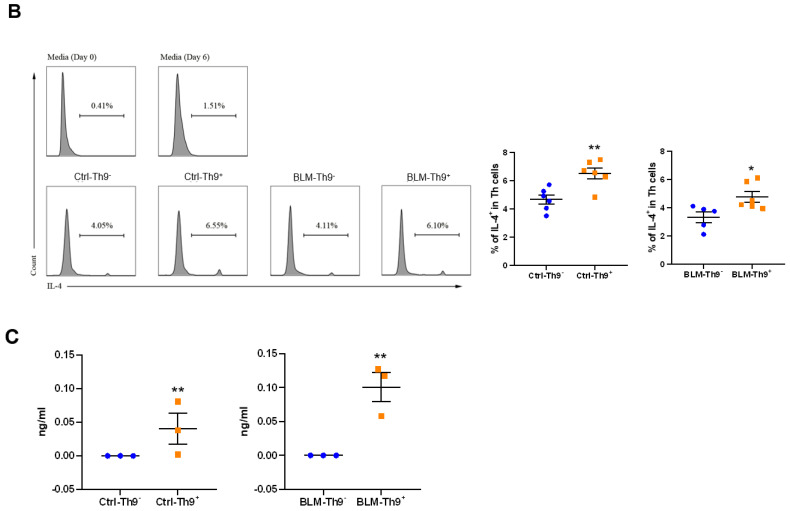
Th9 cells promote Th0 cells (Naive CD4^+^ T cells) differentiating to Th2 cells and induce lung fibroblasts to secrete more collagen. (**A**) Flowchart of the co-culture assay. (**B**) Co-culture of Th9 cells and Th0 cells promotes the differentiation of Th0 cells to Th2 cells (*n* = 5 to 6). (**C**) Co-culture of Th9 cells and lung fibroblasts induces fibroblasts to secrete more collagen (*n* = 3). *p* values were determined by two-sided Student’s *t*-test. Data were expressed as mean ± SEM. * *p* < 0.05; ** *p* < 0.01.

We next explored the effects of Th9 cells on lung cells. To test the effect of Th9 cells on Th0 cells in vitro, CD4^+^ Th0 cells in the lungs of BLM mice and control mice were sorted by flow cytometry and induced into Th9 cells in vitro, and then co-cultured respectively with Th0 cells sorted from normal mice (Figure 4A). 6 days after co-culture, Th2 cell content was detected by flow cytometry. As compared to the cells that were not induced into Th9 cells (Ctrl-Th9^−^ group: 4.660 ± 0.3282%; BLM-Th9^−^ group: 3.326 ± 0.3808%), cells that were induced into Th9 (either derived from normal mice or BLM mice) were found to promote the differentiation of Th0 cells to Th2 cells (Ctrl-Th9^+^ group: 6.523 ± 0.3881%, *p* < 0.01; BLM-Th9^+^ group: 4.782 ± 0.3843%, *p* < 0.05) (Figure 4B). As we know, Th0 cells selectively differentiate into Th1 or Th2 cells in different microenvironments and Th1/Th2 is in a dynamic balance under normal circumstances. The above experiments showed that Th9 cells can promote the differentiation of Th0 cells to Th2 cells, thereby accelerating Th1/Th2 imbalance and forming a positive feedback to promote fibrosis. We then examined the effect of Th9 cells on collagen secretion of cultured fibroblasts. Th9 cells induced in vitro were co-cultured with mouse lung fibroblasts for 6 days and collagen content in the cell supernatant was assayed by ELISA (Figure 4A). As compared to the cells that were not induced, co-culture with the induced Th9 cells (whether derived from normal mice or BLM mice) resulted in a higher collagen content in the cell supernatant, indicating that Th9 cells can induce lung fibroblasts to secrete more collagen in vitro (*p* < 0.01) (Figure 4C).

### 2.5. Preventive Treatment of Neutralizing IL-9 Reduces Pulmonary Fibrosis and Collagen Secretion

**Figure 5:**
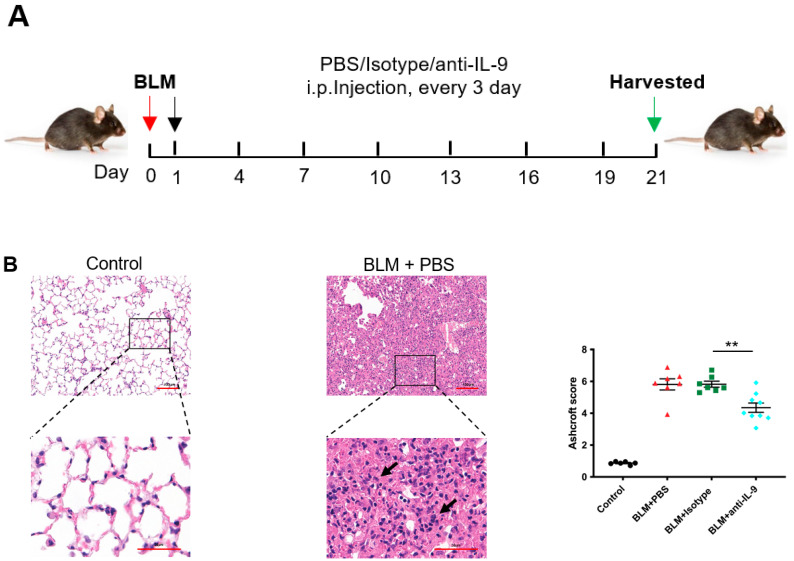

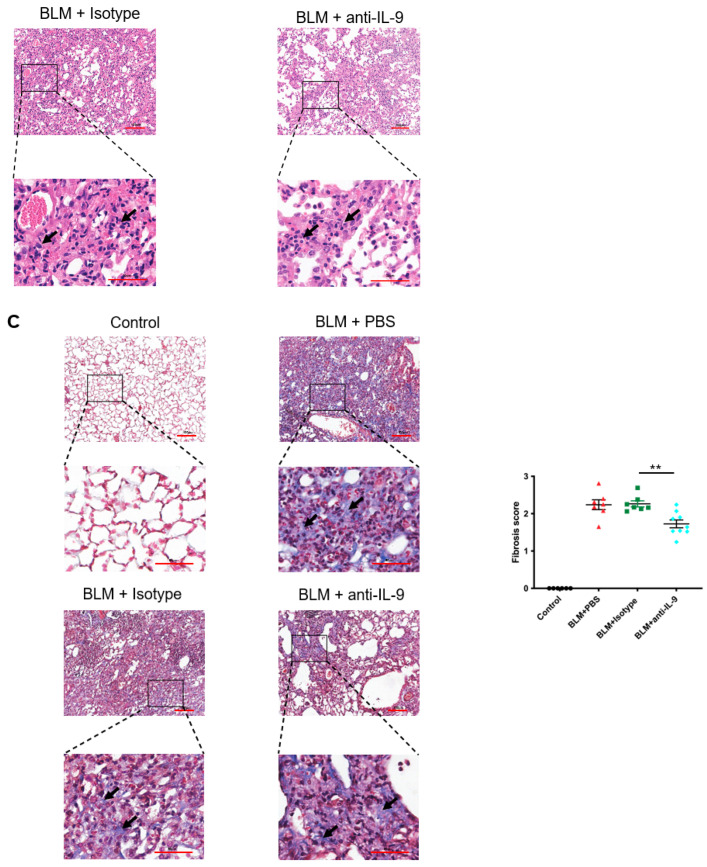

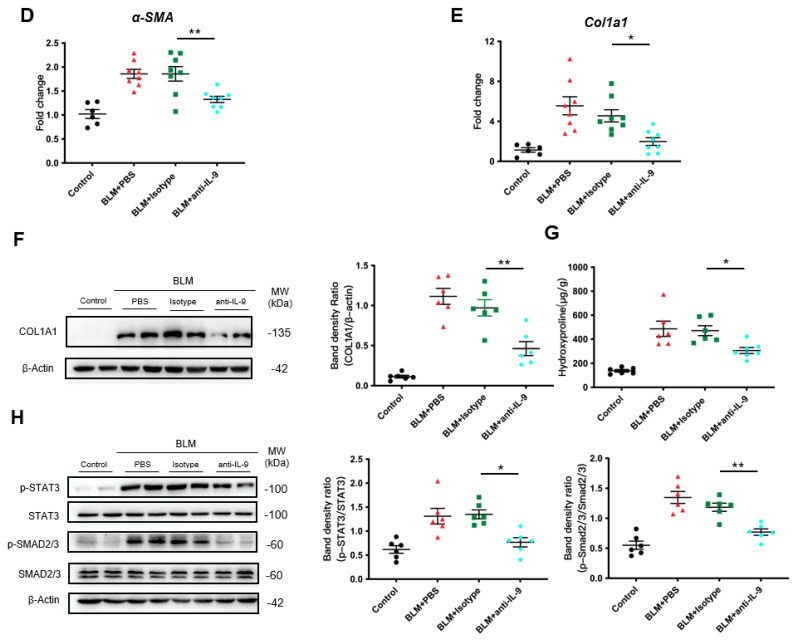
Neutralizing IL-9 attenuates pulmonary fibrosis and collagen content in the bleomycin (BLM)-induced pulmonary fibrosis mice (preventive intervention). (**A**) Schematic showing therapeutic intervention in the BLM model. Drugs (PBS/Isotype/anti-IL-9, every 3 days) were injected intraperitoneally starting at day 1, and lungs were assessed on day 21 after BLM administration. (**B**) Representative HE staining and quantitative analysis of lung sections from mice of different experimental groups (*n* = 6 to 12). Inflammatory cell infiltration was indicated by black arrows. Scale bars, 50 μm. (**C**) Representative Masson’s trichrome staining and quantitative analysis of lung sections from mice of different experimental groups (*n* = 6 to 12). Deposited collagen was indicated by black arrows. Scale bars, 50 μm. (**D**–**E**) Quantitative PCR analysis of *α-SMA* (**D**), *Col1a1* (**E**) (*n* = 6 to 8) in mice of different experimental groups. (**F**) Representative Western blot images and quantitative analysis of COL1A1 in lung homogenates from mice of different experimental groups (*n* = 6). β-Actin was used as a loading control. (**G**) Lung hydroxyproline content in mice of different experimental groups (*n* = 6 to 7). (**H**) Representative Western blot images and quantitative analysis of STAT3 and SMAD2/3 phosphorylation and total expression of STAT3 and SMAD2/3 in lung homogenates from mice of different experimental groups. (**C**–**H**) *p* values were determined by one-way ANOVA. Data were expressed as mean ± SEM. * *p* < 0.05; ** *p* < 0.01.

The above experiments demonstrated that Th9 cells and IL-9 promote pulmonary fibrosis. Next, we verified whether neutralizing IL-9 could reduce pulmonary fibrosis of BLM mice. The BLM model undergoes an inflammatory phase (0–7 d) and a fibrosis phase (after 7 d). Animal drug administration during these two phases is regarded as preventive and therapeutic, respectively [37]. From the first day after BLM instillation, we intraperitoneally injected BLM mice with IL-9 neutralizing antibody every 3 days (Figure 5A). HE staining showed that, 21 days after BLM treatment, the lung tissue of BLM group (BLM + PBS) and IgG isotype control group (BLM + Isotype) had severe damage and inflammatory cell infiltration (indicated by the black arrow). BLM + Isotype group was defined as the control group. The inflammatory response in the lungs of IL-9 neutralizing antibody group was significantly decreased (Figure 5B). The degree of pulmonary fibrosis was evaluated with Ashcroft score (the more severe the degree of fibrosis, the higher the Ashcroft score) and we found that neutralizing IL-9 had remarkable lower Ashcroft scores than control group (*p* = 0.0024) (Figure 5B). Distribution of collagen fibers in the lung tissue and degree of fibrosis were also evaluated by Masson staining (the more severe the degree of fibrosis, the higher the fibrosis score). It was found that 21 days after BLM instillation, collagen deposition in the mice lungs of BLM group and IgG isotype control group markedly increased; whereas IL-9 neutralizing antibody group had less collagen deposition than control group (Figure 5C). Similarily, the fibrosis score of IL-9 neutralizing antibody group was significantly lower than that of control group as well (*p* = 0.0032) (Figure 5C).

In addition, we found that neutralizing IL-9 significantly reduced the expression of *α-SMA* (an indicator for fibroblast activation) in lung tissue by real-time qPCR as compared to control group (*p* = 0.0078), indicating a decreased activation of fibroblasts (Figure 5D). Meanwhile, expression level of *Col1a1* in the treated group was significantly lower than that of control group (*p* = 0.035) (Figure 5E). In addition, protein level of COL1A1 in the treated group was decreased as compared to control group (*p* = 0.0022) (Figure 5F). We also measured the content of hydroxyproline (a major component of collagen) in lung tissue and found a markedly reduced level in the treated group (*p* = 0.0379) (Figure 5G). Neutralizing IL-9 significantly reduced phosphorylation of STAT3 (*p* = 0.0093) and SMAD2/3 (*p* = 0.0048) (Figure 5H). These results demonstrated that neutralizing IL-9 reduces the degree of pulmonary fibrosis and collagen secretion. Moreover, phosphorylation of STAT3 and SMAD2/3 is involved.

### 2.6. Effects of IL-9 Neutralizing Antibody on the Ratio of Th9 Cells, Th2 Cells, and Th1/Th2 in Lung Lymphocytes of BLM Mice in Preventive Treatment

**Figure 6:**
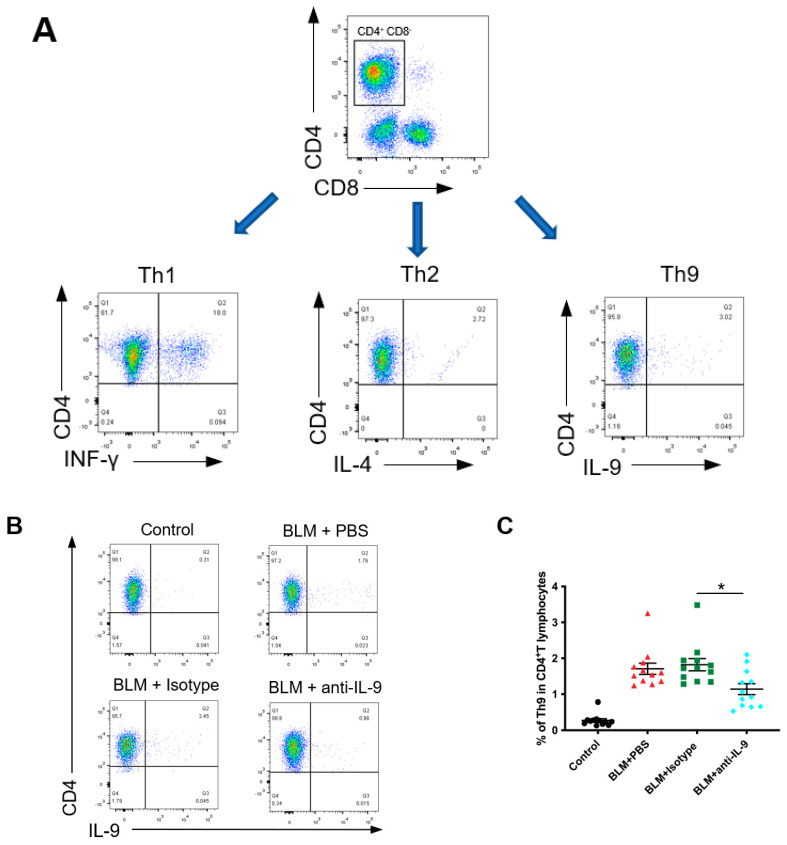

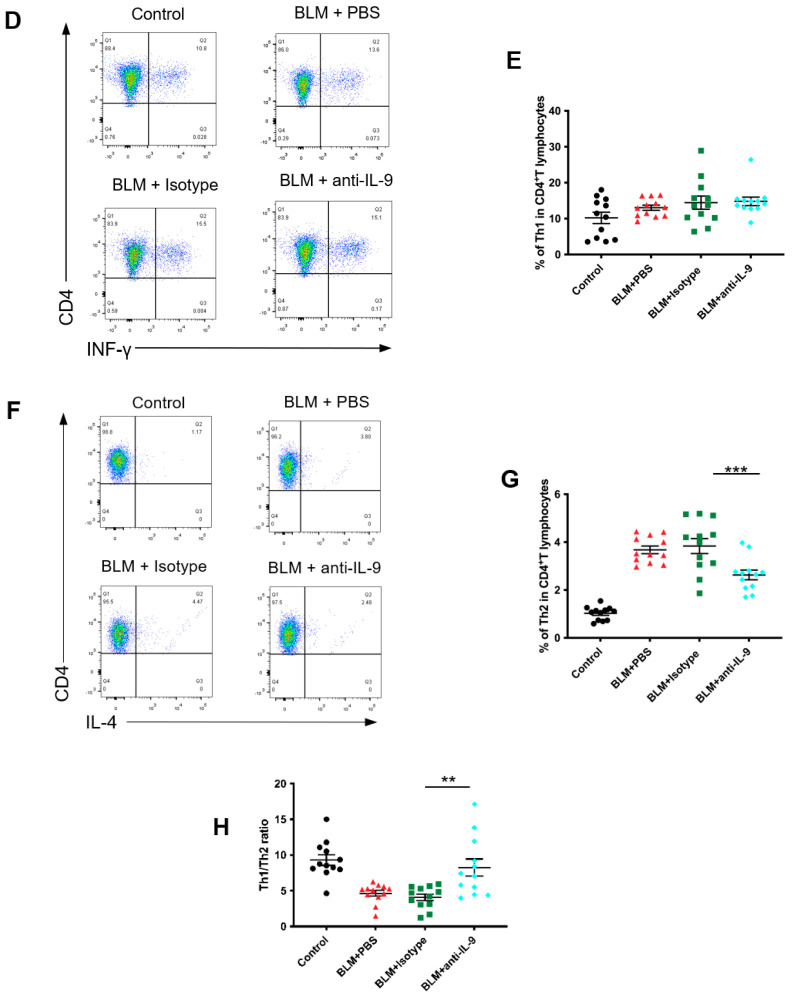
Effects of IL-9 neutralizing antibody on the ratio of Th9 cells, Th2 cells, and Th1/Th2 in lung lymphocytes of BLM-induced pulmonary fibrosis mice (preventive intervention). (**A**) Flow cytometric analysis of INF-γ, IL-4, and IL-9 in the mice lung tissue. Gating strategy was used to identify Th1, Th2, and Th9 cells in the lungs of mice (CD4^+^ IL-9^+^ T cells were defined as Th9 cells, CD4^+^ IFN-γ^+^ T cells were defined as Th1 cells, CD4^+^ IL-4^+^ T cells were defined as Th2 cells). (**B**,**D**,**F**) Representative pseudo-color plot showing proportion of Th9, Th1, and Th2 to CD4^+^ T cells in the mice lung detected by flow cytometry (**C**,**E**,**G**) Proportions of Th9, Th1, and Th2 cells to CD4^+^ T cells in the lungs of mice from different experimental groups. (**H**) Th1/Th2 ratio in the lungs of mice from different experimental groups. *p* values were determined by one-way ANOVA (Tukey’s test). Data were expressed as mean ± SEM. * *p*<0.05, ** *p*<0.01, *** *p*<0.001.

We performed flow cytometry analysis of lung lymphocytes from BLM mice (Figure 6A) and found that IL-9 neutralizing antibody reduced the ratio of Th9 cells in CD4^+^ T cells in the lungs as compared to that of control group (*p* = 0.008) (Figure 6B,C). Does neutralizing IL-9 affect Th1 and Th2 content and Th1/Th2 ratio? We then analyzed the proportion of Th1, Th2, and Th1/Th2 in the lung CD4^+^ T cells of BLM mice, and found no statistical difference in the proportion of Th1 cells among the groups (Figure 6D,E). However, the proportion of Th2 cells in lung CD4^+^ T cells of the IL-9 neutralizing antibody group was markedly lower than that of control group (*p* = 0.001) (Figure 6F,G). In addition, IL-9 neutralizing antibody increased the Th1/Th2 ratio in the lungs as compared to control group (*p* = 0.0021) (Figure 6H). The above results indicated that the imbalance of Th1/Th2 in the lungs of BLM mice is due to an elevated Th2 response. IL-9 neutralizing antibody restores Th1/Th2 balance and reduces pulmonary fibrosis by inhibiting Th2 cell differentiation.

Most clinical drugs that have only been tested in preventive intervention studies proved to be ineffective. Therefore, therapeutic intervention, rather than preventive intervention, is more in line with clinical practice. We started therapeutic intervention on the day 7 after BLM instillation and administered drugs to mice every 3 days to examine whether this intervention could reduce the degree of pulmonary fibrosis (Figure S1A). Similar as what we observed in the preventive study therapeutic treatment of neutralizing IL-9 reduces pulmonary fibrosis and collagen secretion of BLM mice. Phosphorylation of STAT3 and SMAD2/3 is involved as well (Figure S1B–G). Furthermore, IL-9 neutralizing antibody can also restore Th1/Th2 balance (Figure S2).

### 2.7. Proteomics Study Identify Additional Signal Pathways Involved in the Effect of IL-9 Neutralizing Antibody on Pulmonary Fibrosis

**Figure 7:**
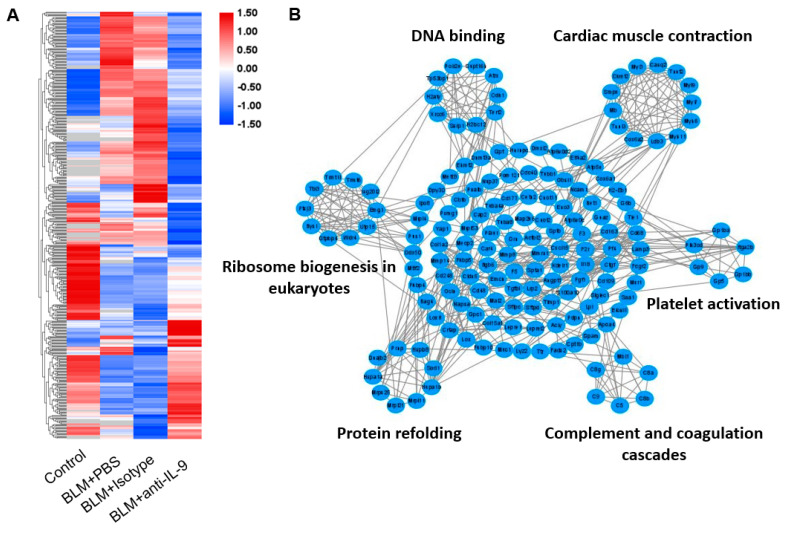

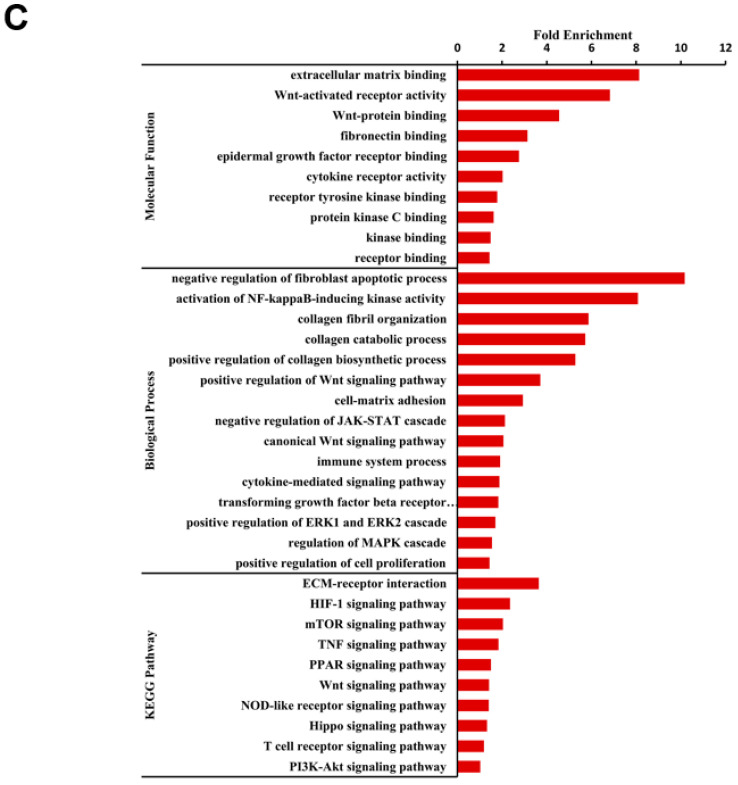

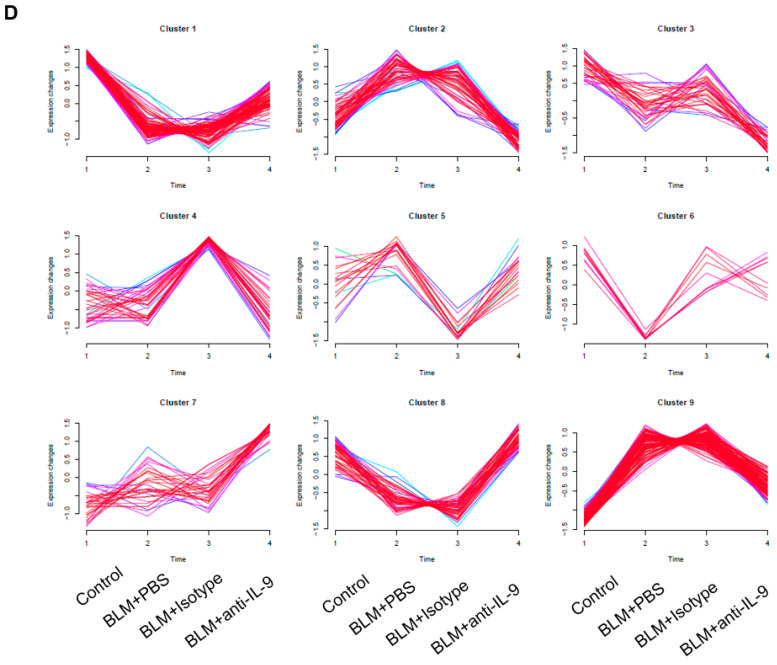
Proteomics analysis of lung homogenates from mice of four groups (therapeutic intervention). (**A**) Heatmap representation of abundance profile of all differentially expressed proteins in four groups. (**B**) Network diagram showing interactions between proteins extracted from STRING database. MCODE method was used to calculate clusters. Six clusters were identified. (**C**) GO-based enrichment and KEGG pathway enrichment analysis of proteins. (**D**) Clustering of proteome expression profiles.

We also conducted a comparative proteomics study in an attempt to identify genes and important signal pathways involved in this intervention. A heatmap represents abundance profile of all differentially expressed proteins in the four groups (Figure 7A). To better understand the function of these differentially expressed proteins, they were subjected to a protein–protein interaction (PPI) network analysis using the STRING database. The PPI network was analyzed for highly connected nodes by MCODE. Six highly connected clusters have been identified including complement and coagulation cascades, protein refolding, ribosome biogenesis in eukaryotes, DNA binding, cardiac muscle contraction, and platelet activation (Figure 7B). We also performed GO-based enrichment and KEGG pathway enrichment analysis of proteins (Figure 7C). Negative regulation of JAK-STAT cascade, regulation of MAPK cascade, cytokine-mediated signaling pathway, PPAR signaling pathway, etc. were enriched. To draw an unbiased picture of the proteomic changes occurring in different groups, we next clustered the 438 differential proteins on the basis of their expression in different interventions into nine distinct profiles (Figure 7D). Cluster 1 (e.g., C9, Fgf1, Yap1, Gp5) and 8 (e.g., C8g, Tgtp1, Tie1, Lamp3) represent those proteins that decline post BLM instillation and increase in the intervention group, whereas cluster 2 (e.g., Vmac, Ppp1r14b, Irgq) and 9 (e.g., Tgfbi, Col15a1, Nfkbie, Timp1) consist of proteins that rise post BLM instillation and decline in the intervention group. These four clusters are of our interest as they include the proteins that have the opposite effect of BLM.

## 3. Discussion

IPF is a chronic, progressive, lethal disease characterized by pulmonary fibrosis that is difficult to cure. Our study showed that Th9 cells have increased differentiation and activation in the lung tissue of patients with IPF and BLM mice. We also found that Th9 cells promote pulmonary fibrosis via IL-9 and IL-4 mediated pathways. Furthermore, IL-9 neutralizing antibody alleviates pulmonary fibrosis.

Disorders of immune regulation play an important role in the pathogenesis of IPF [5]. Previous studies have found that Th9 cells abnormally elevated in bronchial asthma and cystic fibrosis, promoting disease progression [38,39]. We speculated that Th9 cells may also be abnormal and play a deleterious role in pulmonary fibrosis. Our study confirmed this conjecture. We found an increased differentiation and activation of Th9 cells in the lung tissues of IPF patients and BLM mice.

The role of Th9 cells in pulmonary fibrosis is unknown. As we found an elevated level of IL-9 (the main cytokine secreted by Th9 cells) in BALF from BLM mice, we therefore first investigated the in vitro effect of IL-9 on fibroblasts, the major effector cells of pulmonary fibrosis. We used TGF-β (the main fibrogenic growth factor) to simulate the pulmonary fibrosis microenvironment and found that IL-9 promoted the proliferation and activation of mouse primary lung fibroblasts, either alone or in the presence of TGF-β. Previous studies have shown that IL-9 affects a variety of cells, and its role in promoting or inhibiting pulmonary fibrosis varies from cell to cell. On the one hand, IL-9 can recruit mast cells and promote their secretion of TGF-β [20,21]. It can also inhibit the Th1-type immune response partially mediated by antigen-presenting cells and downregulate the expression of anti-fibrotic molecules IL-12 and IFN-γ, having a pro-fibrotic effect [22]. On the other hand, IL-9 can induce monocyte-macrophage differentiation, thereby inducing the synthesis of the anti-fibrotic molecule PGE2 and exerting an anti-fibrotic effect [23]. Previous studies have reported that IL-9 affects the progress of pulmonary fibrosis. However, their findings are inconsistent [24,25,26,27,28,40,41]. In order to clarify the role of IL-9 in pulmonary fibrosis, we administered IL-9 neutralizing antibody to intervene BLM-induced pulmonary fibrosis in mice. Similar to a study by Sugimoto et al. [24], we found that neutralizing IL-9 effectively reduced the degree of pulmonary fibrosis in BLM mice. Both our and Sugimoto’s studies suggested that IL-9 promotes pulmonary fibrosis. Contrary to this conclusion, Arras et al. found that IL-9 overexpression alleviated pulmonary fibrosis by inhibiting Th2 response in bleomycin or silica-induced pulmonary fibrosis mice models [25,41]. Why were their findings inconsistent with our and those of Sugimoto? We noticed that they used Tg5 mice, a transgenic mice overexpressing IL-9. Immune regulation is a complex system, and cytokines form an interconnected and interacting network [42]. The extreme abundance of an individual factor will affect the formation and operation of the entire network system, and such an immune environment will not occur in a real disease state. Hoyle et al. also thought that the Th2 response inhibition of Tg5 mice with IL-9 overexpression and a resulted Th1/Th2 imbalance actually would not occur in lung fibrosis models in the natural state [43]. Therefore, this may be one of the reasons why Arras’ study was different from our study as well as Sugimoto’s.

We analyzed the percentages of various lung lymphocyte subsets of BLM mice by flow cytometry and found that the proportion of CD4^+^IL9^+^IL-4^+^ T cell subset was increased in the lung tissue of BLM mice, suggesting an increased IL-4 secretion by Th9 cells in the lungs of BLM mice. However, whether Th9 cells secrete IL-4 is controversial. By inducing Th0 cells to differentiate into Th9 cells with IL-4 and TGF-β in vitro, Schmitt and Veldhoen did not detect IL-4 content in the cell culture supernatant by ELISA [44,45]. In contrast, Saeki’s study showed that Th9 cells have the ability to secrete IL-4 [29]. IL-4 can stimulate Th0 cells to differentiate into Th2 cells [46]. We then hypothesized that Th9 cells may promote the differentiation of Th0 cells to Th2 cells by secreting IL-4, thereby accelerating the Th1/Th2 imbalance and forming a positive feedback to promote fibrosis process. Therefore, we co-cultured in vitro differentiated Th9 cells with Th0 cells and found that Th9 cells indeed promoted the differentiation of Th0 cells to Th2 cells. In addition, IL-4 can also promote fibroblast proliferation and activation [10,47]. We then examined the effects of Th9 cells on fibroblasts in vitro and found that Th9 cells promoted collagen secretion. This may be due to the combined effect of IL-4 and IL-9 secreted by Th9 cells (we have already shown that IL-9 promotes fibroblast activation and collagen secretion). Collectively, these results indicate that Th9 cells promote fibrosis in vitro.

Our results also showed that IL-9 neutralizing antibody can reduce the proportions of Th2 cells and Th9 cells in the lungs of BLM mice. Arendse et al. found that neutralizing IL-9 reduced Th2 response in mice [48]. Munitz et al. reported that IL-9 can recruit Th2 cells through mast cells [49]. These studies indicated that neutralizing IL-9 in vivo may reduce the Th2 proportion. Moretti et al. found that IL-9 increased IL-2 production by mast cells, resulting in an expansion of CD25^+^ type 2 congenital lymphoid cells (ILC2) and subsequent activation of Th9 cells, suggesting that neutralizing IL-9 may reduce Th9 cell activation by blocking this pathway [39]. Cai et al. also showed that neutralizing IL-9 reduced the proportion of Th9 cells in mice [50]. Consistent with these findings, we found that neutralizing IL-9 reduced the proportions of Th2 cells and Th9 cells in the lungs of mice.

Activation of STAT3 participates in fibrosis pathways, leading to lung fibrosis by promoting epithelial damage and fibroblast activation [51,52]. Smad family contains a series of structurally similar proteins, which are the main signal transducers of TGF-β superfamily receptors and essential for regulating cell development and growth [53]. SMAD2/3 participates in the pulmonary fibrosis pathway, and targeting TGF-β-mediated SMAD2/3 signaling prevents fibrosis [36]. Our results suggest that neutralizing IL-9 may alleviate pulmonary fibrosis by regulating the STAT3 and SMAD2/3 pathways.

Interventions in BLM model are usually divided into preventive and therapeutic [37]. We first tested preventive intervention from day 1 after the animal model was established. Intratracheal administration of BLM in mice caused a strong inflammatory response in the lungs. Prophylactic administration of IL-9 neutralizing antibody in mice exerted an anti-fibrotic effect via reducing lung inflammation in the early stage (inflammatory phase) and inhibiting extracellular matrix secretion by the (myo)fibroblasts in the late stage (fibrotic phase). Our results showed that IL-9 neutralizing antibody could also play a role in therapeutic intervention (Supplementary Figures).

For the first time, our study reported the deleterious role and underlying mechanism of Th9 cells in patients with IPF and BLM mice, which is different from regarding Th9/IL-9 as a whole in previous studies. We clarified the pro-fibrotic effect of IL-9 and studied its molecular mechanism. In addition, we discovered that Th9 cells also promote pulmonary fibrosis via increasing IL-4-mediated Th2 cell differentiation. Moreover, neutralizing IL-9 effectively reduced the degree of pulmonary fibrosis. One of the limitations of this study, same as many other studies, is that the histological changes, morbidity, or mortality of BLM mice are not completely similar to IPF, although it is a classic model for studying pulmonary fibrosis. Besides, there is currently no specific Th9 cell blocker that enables us to examine the effect of blocking Th9 cells on lung fibrosis in vivo.

In summary, we first elucidated the role of Th9 cells in promoting pulmonary fibrosis through the secretion of IL-9 and IL-4. Moreover, we showed that IL-9 neutralizing antibody ameliorated the degree of pulmonary fibrosis. Our study illustrates the pathogenesis of pulmonary fibrosis from an immunological perspective. In recent years, with the great success of CTLA-4 and PD-1 inhibitors, immunotherapy has played an increasingly important role [54,55]. At present, more and more immunologic clinical studies are ongoing [56,57,58]. In addition, many basic studies have also found that immunotherapy can effectively reduce pulmonary fibrosis and has potential for clinical applications [59,60]. Our findings suggest that Th9-based immunotherapy may be employed as a treatment strategy for IPF.

## 4. Materials and Methods

### 4.1. Reagents and Antibodies

The reagents and antibodies used in this study were listed in Table 3 and Table 4, respectively.

### 4.2. Human Subjects

Lung tissue of IPF patients obtained from surgical lung biopsies, used for immunohistochemistry (*n* = 14), were from The First Affiliated Hospital of Guangzhou Medical University (Guangzhou, China). The IPF patients were diagnosed according to the 2018 ATS/ERS/JRS/ALAT guideline diagnostic criteria [1]. There were 12 males and 2 females, and the mean age was 58.86 ± 8.75 years old. Control samples were paracancerous tissue of patients with lung cancer (*n* = 4). This study was reviewed and approved by the Ethics Committee of The First Affiliated Hospital of Guangzhou Medical University.

### 4.3. Bleomycin-Induced Pulmonary Fibrosis Mice Model and Intervention Study

All animal experiments were approved by the Institutional Animal Care and Use Committee (IACUC) of Guangzhou Medical University. Male C57BL/6J mice (8–9 weeks old) were purchased from Hua Fu Kang company (Beijing, China). Intratracheal administration and BALF collection were performed as previously described [60]. In brief, single intratracheal instillation of saline (50 μL) or BLM (2 mg/kg, dissolved in 50 μL saline) was administrated to the mice. Mice were randomized into four experimental groups (saline + PBS group, BLM + PBS group, BLM + Isotype group, BLM + anti-IL-9 group). In the prevention intervention study, mice were administered drugs via intraperitoneal injections on day 1, 4, 7, 10, 13, 16, 19. In the therapeutic intervention study, mice were injected with drugs on day 7, 10, 13, 16, 19. In both cases, lungs were harvested on day 21 post bleomycin challenge. At the designate time point after BLM instillation, mice were euthanized with pentobarbital. The BALF and lungs were harvested for further analysis.

### 4.4. CCK Assay

Cell Counting Kit-8 (CCK-8) assay (Yeasen) was used to determine the viability of primary mouse lung fibroblasts. Cells were seeded overnight in a 96-well plate at a density of 2 × 10^3^ cells per well, and stimulated with IL-9 (10 ng/mL) or TGF-β (5 ng/mL) for 24 h and 48 h, respectively. 10 μL CCK-8 solution was mixed with 90 μL DMEM/F12 and added to each well. After incubating for 3 h, the absorbance was measured at a wavelength of 450 nm.

### 4.5. Histology and Fibrosis Assessment

Left lungs of the mice were fixed in 4% paraformaldehyde formalin for 24 h and embedded in paraffin. Slices were stained with hematoxylin/eosin (H&E) and Masson trichrome according to standard procedures. Ashcroft score and fibrosis score were used to quantify the degree of lung fibrosis [61,62]. Readers blinded to the treatments randomly and non-repetitively scored 100 fields under a ×20 objective. Then the total score was averaged. According to the score table, the Ashcroft score is between 0 and 8, while the Masson fibrosis score is between 0 and 3.

### 4.6. Hydroxyproline Assay

Hydroxyproline content in lung tissue was determined by hydroxyproline assay kit (Nanjing Jiancheng Bioengineering Institute, Nanjing, China) as previously described (2). In brief, lung tissue was dissolved in sodium hydroxide at 95 °C for 20 min. Hydrolysate was oxidized by chloramine T. Then, Dimethyl-amino-benzaldehyde was added to the suspension to form a compound and the OD value of the compound was detected at 550 nm. The hydroxyproline content in the lung tissue was calculated from the OD value using a standard curve and expressed as microgram per gram (μg/g) of lung tissue.

### 4.7. RNA Extraction and Quantitative Real-Time PCR (qRT-PCR)

Total RNA was extracted from lung tissues or cells using TRIzol reagent (Invitrogen, Carlsbad, CA, USA) according to the manufacturer’s protocol. Total RNA was reverse transcribed with PrimeScript^TM^ RT reagent Kit with gDNA Eraser (Perfect Real Time) (Takara, Otsu, Japan) and qRT-PCR was performed in triplicate using TB Green^®^ Premix Ex Taq^TM^ II (Tli RNaseH Plus) (Takara, Otsu, Japan) on the Biosystems^®^ QuantStudio^TM^ 5 Flex Real-Time PCR System (Thermo Fisher Scientific, Waltham, MA, USA). GAPDH was used as an endogenous control. Relative RNA expression levels were calculated using the 2^−ΔΔCt^ method. The primer sequences are listed in Table 5.

### 4.8. Flow Cytometry

#### 4.8.1. Human

Human peripheral blood mononuclear cells (PBMC) were isolated from blood samples using Ficoll–Paque Plus (GE Healthcare, Pittsburgh, PA, USA). Isolated cells were incubated with phorbol 12-myristate 13-acetate (PMA) (50 ng/mL; Abcam, Cambridge, MA, USA), ionomycin (1 μg/mL; Abcam, Cambridge, MA, USA) and GolgiPlug (1 μg/mL, BD Biosciences, San Diego, CA, USA) for 6 h at 37 °C under a 5% CO_2_ atmosphere. Next, cells were transferred to flow cytometry tubes (2 × 10^6^ cells per tube) and blocked with Human BD Fc Block^TM^ (BD Biosciences, San Diego, CA, USA) for 10 min at room temperature. PerCP-Cy5-conjugated anti-human CD3 (BioLegend, San Diego, CA, USA), Brilliant Violet 510-conjugated anti-human CD8 (BioLegend, San Diego, CA, USA) and Alexa 488-conjugated anti-human CD4 (BD Biosciences, San Diego, CA, USA) antibodies were added to the cells, and incubated for 30 min in the dark at 4 °C. After fixation and permeabilization (BD Biosciences, San Diego, CA, USA), cells were intracellularly stained with Alexa Fluor^®^ 647 mouse anti-human IL-9 antibody (BD Biosciences, San Diego, CA, USA) and incubated for 30 min in the dark at 4 °C. Th9 cells were defined as CD4^+^IL-9^+^ T cells.

#### 4.8.2. Mouse

The lung tissue of the mice was digested with collagenase and gently dispersed through a 70 μm cell strainer (BD Falcon) to prepare a single cell suspension, and the lung lymphocytes were separated by mouse lymphocyte separation medium (DAKEWE, Shenzhen, China). The isolated cells wash twice with Hanks’ Balanced Salt Solution and diluted to a concentration of 2 × 10^6^ cells/mL in RPMI 1640 culture medium supplemented with 10% heat-inactivated FBS and penicillin/streptomycin (Gibco, Grand Island, NY, USA). Cells were stimulated by PMA (50 ng/mL; Abcam, Cambridge, MA, USA) and ionomycin (1μg/mL; Abcam, Cambridge, MA, USA) for 1 h, and incubated with Brefeldin A (1 μg/mL; Abcam, Cambridge, MA, USA) for 4 h to avoid cytokine secretion. Then, anti-mouse CD16/32-V450 antibody (eBioscience, San Diego, CA, USA) was added into the cells at room temperature for 10 min. Then, APC-Cy7-conjugated anti-mouse CD3, PE-Cy5-conjugated anti-mouse CD4 and FITC-conjugated anti-mouse CD8 (BD Biosciences, San Diego, CA, USA) antibodies were added into the cells, and incubated for 30 min in the dark at 4 °C. After surface staining, the cells were intracellularly stained with anti-mouse IL-9-PE, anti-mouse IL-4-PE and INF-γ-APC antibodies (BD Biosciences, San Diego, CA, USA) after fixation and permeabilization. Th9, Th2, and Th1 cells were defined as IL-9^+^CD4^+^, IL-4^+^CD4^+^, and IFN-γ^+^CD4^+^ T cells respectively.

### 4.9. Isolation and Culture of Primary Fibroblasts

Primary fibroblasts were isolated from C57BL/6J mice as previously described [63] and were cultured in DMEM/F12 supplemented with 10% FBS and 1% antibiotics in 5% CO_2_ at 37 °C in a humidified atmosphere. Cells of passages 4–6 were used for experiments.

### 4.10. Western Blot Analysis

The mouse lung tissues and cells were homogenized in RIPA lysis buffer containing protease and phosphatase inhibitors (Sigma, P5726, P0044, St. Louis, MO, USA). The protein concentration was determined using the BCA Protein Assay Kit (Thermo Fisher Scientific, 23227, Waltham, MA, USA) and mixed with 5X Protein Loading Buffer (Beyotime, P0015L, Shanghai, China), followed by boiling at 95 °C for 10 min. Proteins samples were separated by electrophoresis on a 10% sodium dodecyl sulfate-polyacrylamide gel and transferred to polyvinylidene fluoride (PVDF) membranes. After blocking, membranes were incubated with the primary antibodies (Table 4) overnight at 4 °C followed by HRP-conjugated secondary antibodies (Gsebio, JC-PB001H, JC-PB002H, Xi’an, China). Immunoblots were detected using ECL Western blotting substrate (Thermo Fisher Scientific, 34580, Waltham, MA, USA) and visualized using Tanon-5200 System (Tanon, Shanghai, China). Protein band intensity was quantified using ImageJ software (NIH, Bethesda, MA, USA).

### 4.11. Co-Culture

Mouse lungs were harvested 3 weeks after BLM or saline instillation and prepared for lymphocyte single cell suspension as described above. After surface staining with CD3 and CD4, Th0 cells (CD3^+^CD4^+^ cells) were sorted by flow cytometry as described above and then induced to Th9 cells (CD3^+^CD4^+^IL-9^+^ cells) by stimulating with IL-4 and TGF-β for six days. These cells were co-cultured with freshly isolated mouse Th0 cells or lung fibroblasts at a 1:1 ratio (Figure 4A). Th2 cells (CD3^+^CD4^+^IL-4^+^ cells) proportion and collagen content were determined by flow cytometry and ELISA, respectively.

### 4.12. Immunofluorescence and Image Analysis

Lung fibroblasts were seeded on a slide in 24-well plate at a density of 5 × 10^4^ cells per well. Cells were fixed in cold 4% paraformaldehyde and permeabilized with 0.5% Triton X-100 in PBS. After blocking with 0.5% BSA and 0.1% Tween-20 in PBS, cells were incubated with primary antibody against α-SMA (ab5694, Abcam, Cambridge, MA, USA), followed by FITC-conjugated secondary antibody (ab6717, Abcam, Cambridge, MA, USA). DAPI was used as a nuclear counterstain. Images were acquired with an Operetta high-content imaging system IX83 (Olympus). Fluorescence intensity for α-SMA staining was analyzed by Image J software (NIH, Bethesda, MA, USA).

### 4.13. Mass Spectrometry and Data Analysis

In brief, mouse lung tissues were ground, lysed, and digested, respectively. Equal amounts of peptides from each sample were fractionated by offline basic pH reverse phase liquid chromatography (LC). Fractionated peptides were analyzed by LC-MS/MS. Using the MaxQuant software, spectra were searched against the Mus_musculus_10090 (17045 sequences) concatenated with reverse decoy database and filtered to achieve 1% FDR at either unique protein level. To minimize redundancy, protein identifications from shared peptide sequences were grouped into unique proteins according to the principal of parsimony. LFQ intensities were extracted, filtered, normalized, and summarized into peptide and protein quantification. One-way ANOVA was used to identify DE events. To define Raptor-dependent events, z-score-based DE analysis was performed, in which proteins with significant z scores (1.96 as cutoff) were overlapped in two experiments. Principal-component analysis and hierarchical clustering were performed using R (v.3.0.1).

### 4.14. Statistical Analysis

Statistical analysis was performed by using two-sided unpaired Student’s *t*-test. Results are expressed as mean ± standard error of the mean (SEM) or mean ± standard deviation (SD). One-way analysis of variance (ANOVA), followed by Bonferroni’s or Tukey’s multiple comparisons test, was used when comparing more than two sets of data. Correlation analysis was determined by Pearson’s correlation test. All data were analyzed using SPSS 25.0 software (SPSS Inc., Chicago, IL, USA). For all comparisons, *p* < 0.05 was considered statistically significant.

## Figures and Tables

**Table 1:** Demographic and clinical characteristics of healthy controls and patients with IPF (Th9 cells in PBMC).

Characteristic	Patients with IPF (*n* = 16)	Healthy Controls (*n* = 19)	*p* Value
Age (years)	64.13 ± 6.62	65.45 ± 7.39	0.60
Male, *n* (%)	16 (100%)	19 (100%)	-

PBMC: peripheral blood mononuclear cell. Data are presented as *n* (%), mean ± SD.

**Table 2:** Demographic and clinical characteristics of controls and patients with IPF (lung tissue immunohistochemistry).

Characteristic	Patients with IPF (*n* = 14)	Controls (*n* = 4)	*p* Value
Age (years)	58.86 ± 8.75	60.5 ± 3	0.72
Male, *n* (%)	12 (85.71%)	4 (100%)	-
FEV1 (%pre)	88.43 ± 8.75	94.25 ± 5.06	0.15
FVC (%pre)	73.21 ± 5.96	88.5 ± 3	<0.001 ***
DL_CO_ (%pre)	62.43 ± 6.38	87.25 ± 3.77	<0.001 ***

Data are presented as *n* (%), mean ± SD. FVC (% predicted), forced vital capacity (% predicted); FEV1 (% predicted), forced expiratory volume in 1 s (% predicted); DL_CO_ (% predicted), carbon monoxide diffusing capacity (% predicted). *** *p* < 0.001.

**Table 3:** Reagent information.

Reagent	Source	Catalog#
LEAF^TM^ Purified anti-mouse IL-9 antibody	Biolegend	504802
Ultra-LEAF^TM^ Purified Armenian Hamster IgG Isotype Control	Biolegend	400940
Recombinant Mouse IL-9	R&D Systems	409-ML
Recombinant Mouse TGF-beta 1	R&D Systems	7666-MB
Recombinant Mouse IL-4	R&D Systems	404-ML
Mouse cross linked C-telopeptide of type I collagen (CTX-I) ELISA Kit	CUSABIO	CSB-E12782m
TB Green^®^ Premix Ex Taq^TM^ II (Tli RNaseH Plus)	Takara	RR820A
PrimeScript^TM^ RT reagent Kit with gDNA Eraser (Perfect Real Time)	Takara	RR047A
Bleomycin (BLM)	Hanhui Pharmaceuticals	

**Table 4:** Antibody information.

Primary Antibody	Application/Dilution	Species	Source (Catalog#)
PU.1	ICH (1:200)	Human	Abcam (ab76543)
IL-9	ICH (1:200)	Human	Abcam (181397)
α-SMA	WB (1:1000), IF (1:200)	Rabbit	Abcam (ab5694)
COL1A1	WB (1:500)	Rabbit	Abclonal (A1352)
STAT3	WB (1:1000)	Rabbit	Cell Signaling Technology (4904)
p-STAT3	WB (1:1000)	Rabbit	Cell Signaling Technology (9145)
SMAD2/3	WB (1:1000)	Rabbit	Cell Signaling Technology (8685)
p-SMAD2/3	WB (1:1000)	Rabbit	Cell Signaling Technology (8828)
β-actin	WB (1:5000)	Mouse	EASYBIO (BE0021-100)

**Table 5:** Primer sequence.

Gene	Mouse Primer Sequence(F/R)
*Col1a1*	CCCGTTGGCAAAGATGGTAG
	ACCTTGGCTACCCTGAGAAC
*α-SMA*	GCTGGTGATGATGCTCCCA
	GCCCATTCCAACCATTACTCC
*PU.1*	GTTCTCGTCCAAGCACAAGG
	TTCTTCACCTCGCCTGTCTT
*Irf4*	AGACCAGACTTGCAAGCTCT
	CACCAAAGCACAGAGTCACC
*Gapdh*	AACGACCCCTTCATTGACCT
	CATTCTCGGCCTTGACTGTG

## Data Availability

Not applicable.

## Supplementary Materials

The following are available online at https://www.mdpi.com/article/10.3390/cells10113209/s1, Figure S1: Neutralizing IL-9 attenuates pulmonary fibrosis and collagen content in the bleomycin (BLM)-induced pulmonary fibrosis mice (therapeutic intervention). (**A**) Schematic showing therapeutic intervention in the BLM model. Drugs (PBS/Isotype/anti-IL-9, every 3 days) were injected intraperitoneally starting at day 7, and lungs were assessed on day 21 after BLM administration. (**B**) Representative HE staining and quantitative analysis of lung sections from mice of different experimental groups (*n* = 6 to 8). Inflammatory cell infiltration was indicated by black arrows. Scale bars, 50 μm. (**C**) Representative Masson’s trichrome staining and quantitative analysis of lung sections from mice of different experimental groups (*n* = 6 to 8). Deposited collagen was indicated by black arrows. Scale bars, 50 μm. (**D**) *Col1a1* in mice of different experimental groups (*n* = 6 to 8). (**E**) Representative western blot images and quantitative analysis of COL1A1 in lung homogenates from mice of different experimental groups (*n* = 6). β-Actin was used as a loading control. (**F**) Lung hydroxyproline content in mice of different experimental groups (*n* = 6 to 7). (**G**) Representative images and quantitative analysis of western blots of STAT3 and SMAD2/3 phosphorylation and their total expression in lung homogenates from mice of different experimental groups. (**B**–**G**) *p* values were determined by one-way ANOVA. Data were expressed as mean ± SEM. *, *p* < 0.05; **, *p* < 0.01, ***, *p* < 0.001. Figure S2: Effects of IL-9 neutralizing antibody on the ratio of Th9 cells, Th2 cells, and Th1/Th2 in lung lymphocytes of bleomycin (BLM)-induced pulmonary fibrosis mice (therapeutic intervention). (**A**,**C**,**E**) Representative pseudo-color plot showing proportion of Th9, Th1, Th2 to CD4^+^T cells in the mice lung detected by flow cytometry. (**B**,**D**,**F**) Proportions of Th9, Th1, Th2 cells to CD4^+^T cells in the lungs of mice from different experimental groups. (**G**) Th1/Th2 ratio in the lungs of mice from different experimental groups. *p* values were determined by one-way ANOVA (Tukey’s test). Data were expressed as mean ± SEM. *, *p* < 0.05, **, *p* < 0.01, ***, *p* < 0.001.
