# Supplementary material for: Deleterious Role of Th9 Cells in Pulmonary Fibrosis
Source: Cells. 2021 Nov 17;10(11):3209. doi: 10.3390/cells10113209 (PMC8621886; doi:10.3390/cells10113209)
Supplement: Supplementary file 1 [file cells-10-03209-s001.zip › cells-1405421-supplementary.pptx]

## Slide 1
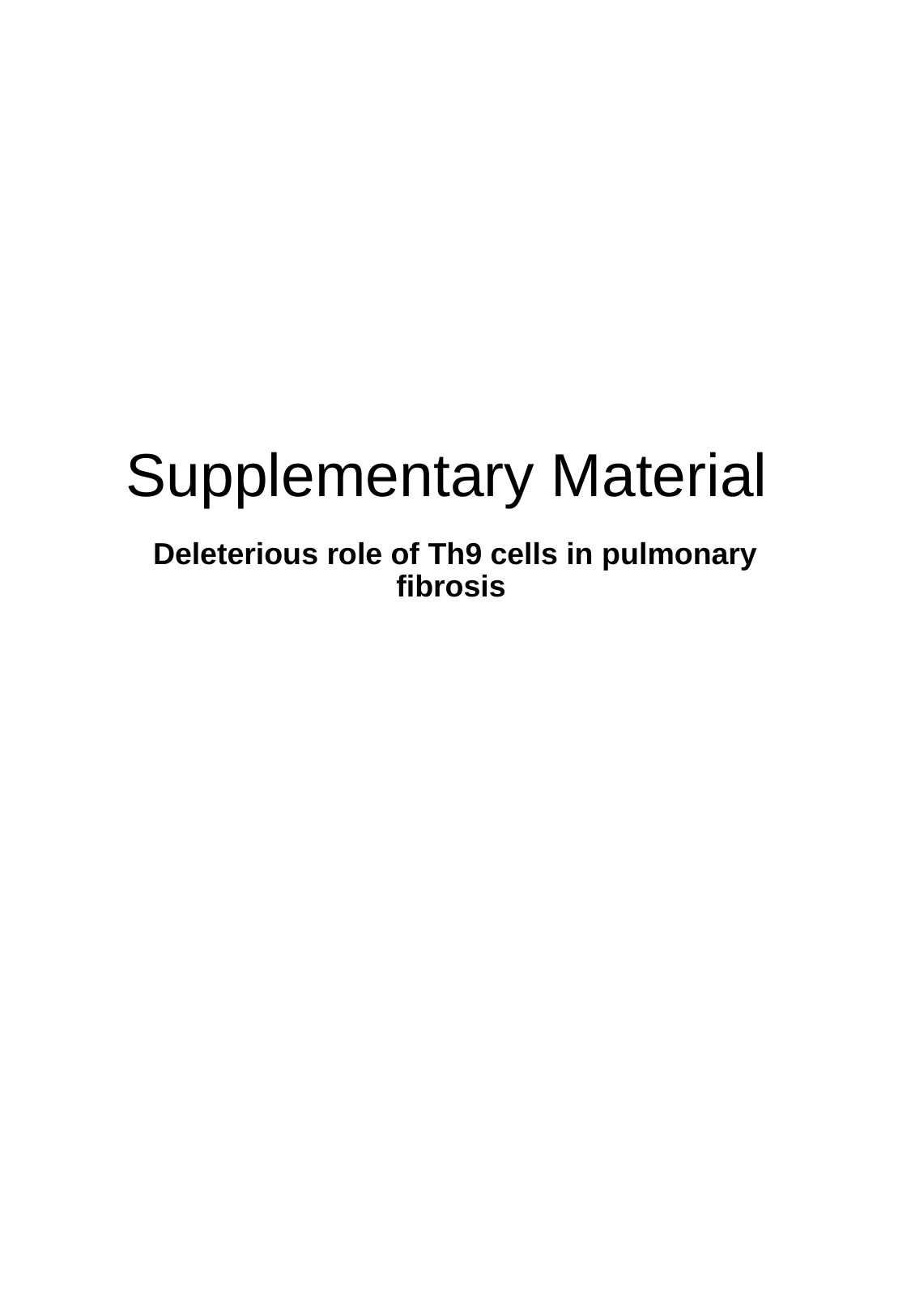

# Supplementary Material
Deleterious role of Th9 cells in pulmonary fibrosis

## Slide 2
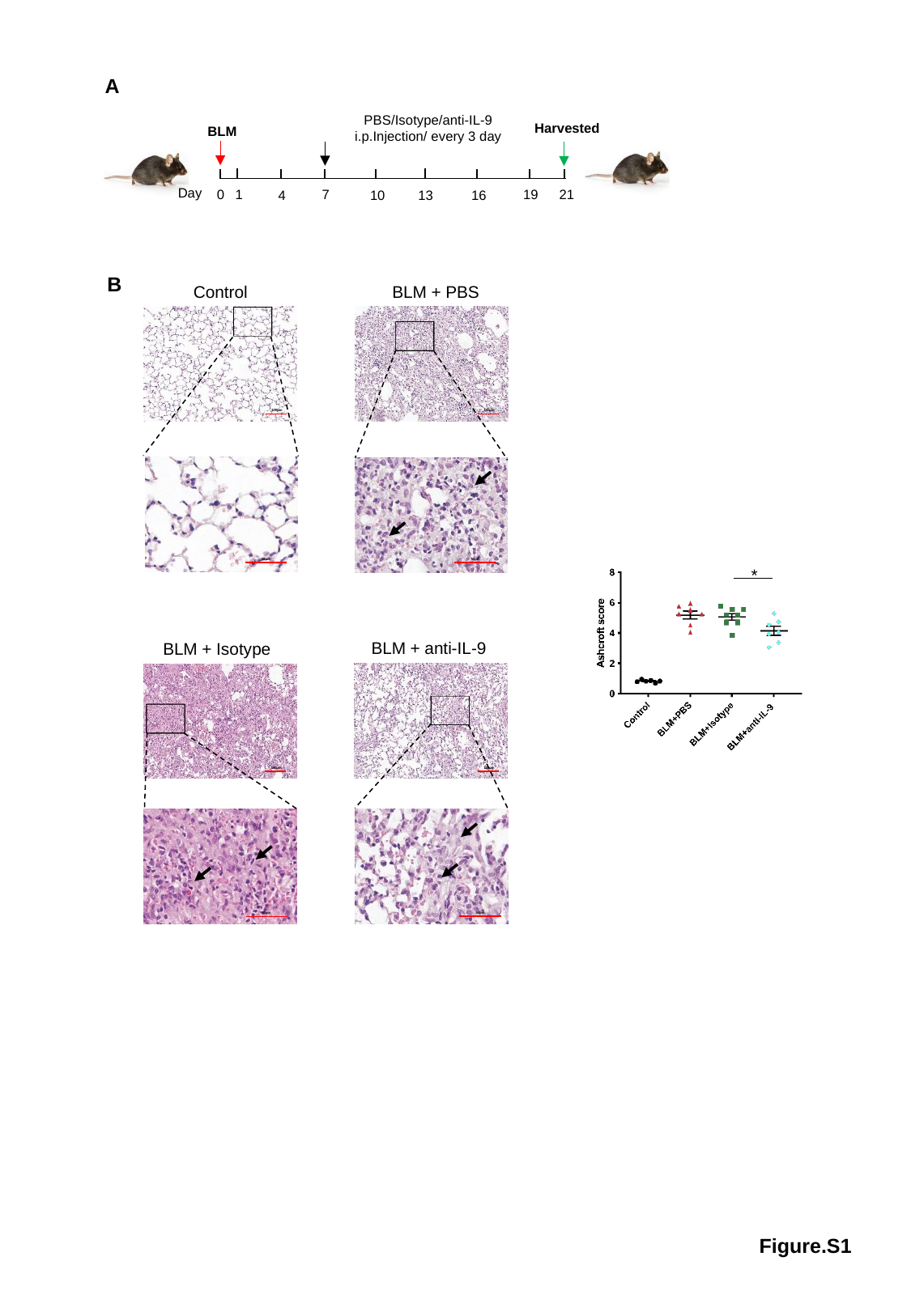

A
PBS/Isotype/anti-IL-9
i.p.Injection/ every 3 day
Harvested
BLM
Day
0
7
1
21
19
16
4
13
10
B
Control
BLM + PBS
100μm
100μm
50μm
50μm
*
BLM + anti-IL-9
BLM + Isotype
100μm
100μm
50μm
50μm
Figure.S1

## Slide 3
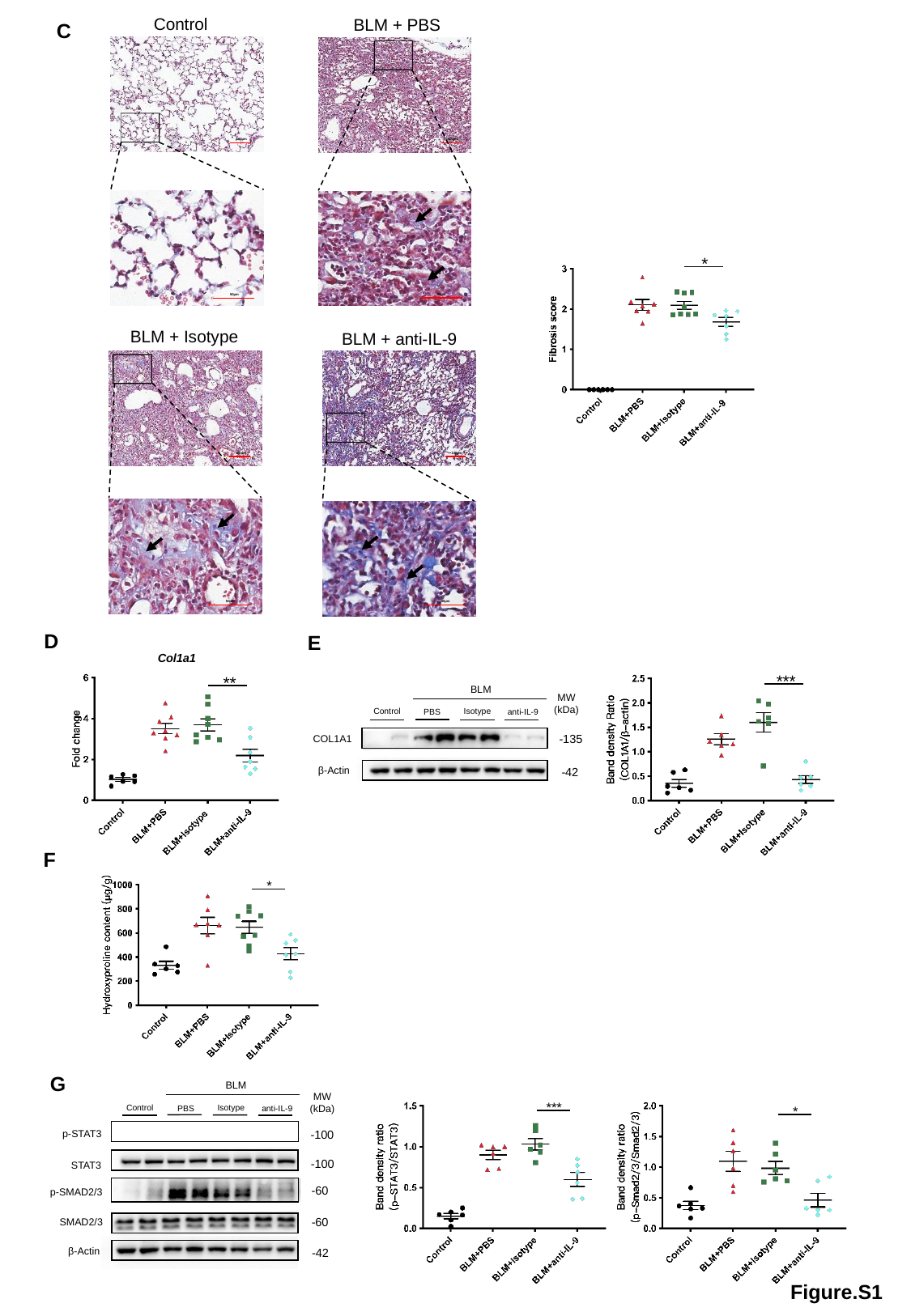

Control
BLM + PBS
C
100μm
100μm
*
50μm
50μm
BLM + Isotype
BLM + anti-IL-9
100μm
100μm
50μm
50μm
D
E
Col1a1
***
**
BLM
MW
(kDa)
Isotype
Control
PBS
anti-IL-9
-135
COL1A1
β-Actin
-42
F
*
G
BLM
MW
(kDa)
***
Isotype
*
Control
PBS
anti-IL-9
-100
p-STAT3
-100
STAT3
-60
p-SMAD2/3
-60
SMAD2/3
β-Actin
-42
Figure.S1

## Slide 4
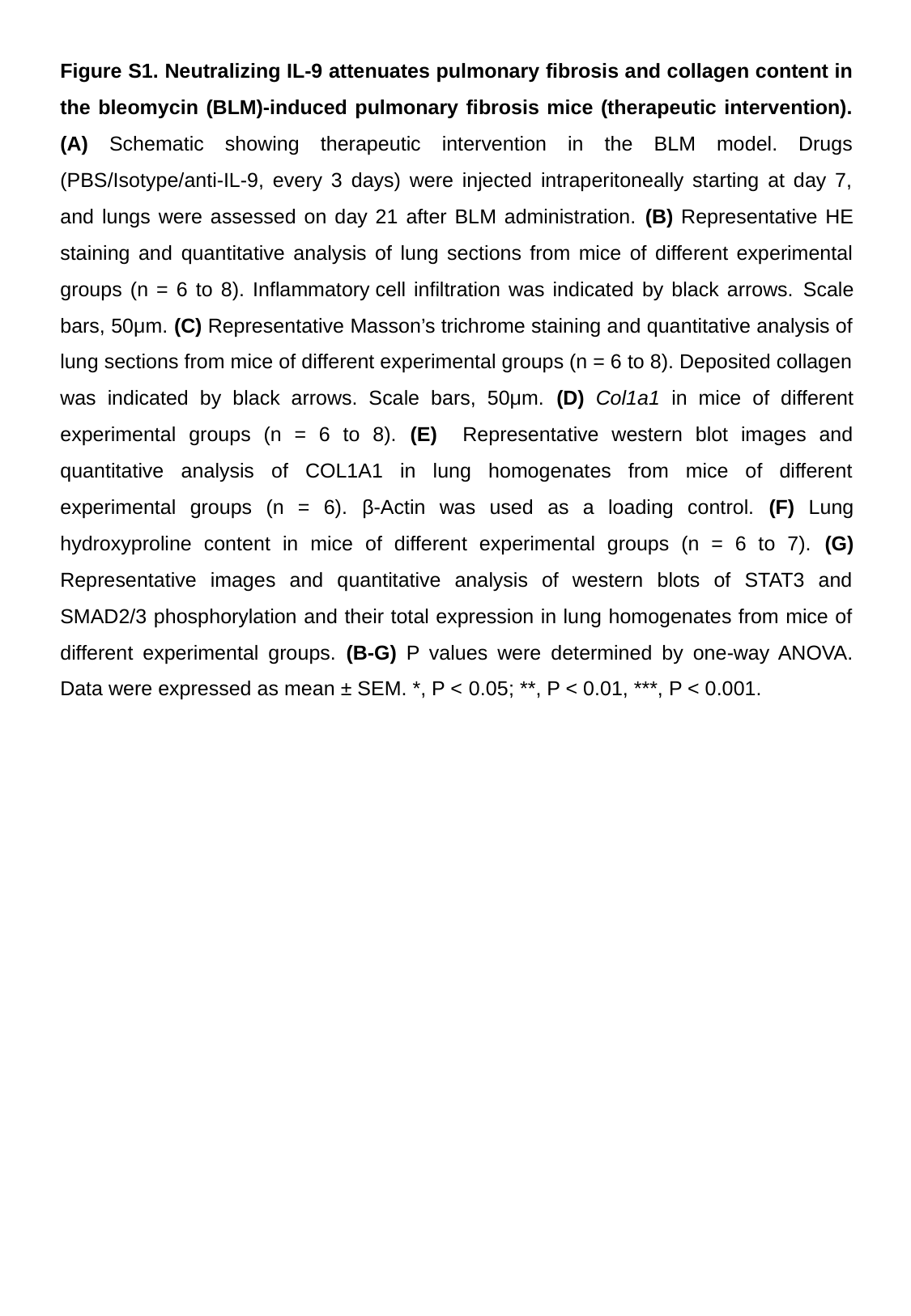

Figure S1. Neutralizing IL-9 attenuates pulmonary fibrosis and collagen content in the bleomycin (BLM)-induced pulmonary fibrosis mice (therapeutic intervention). (A) Schematic showing therapeutic intervention in the BLM model. Drugs (PBS/Isotype/anti-IL-9, every 3 days) were injected intraperitoneally starting at day 7, and lungs were assessed on day 21 after BLM administration. (B) Representative HE staining and quantitative analysis of lung sections from mice of different experimental groups (n = 6 to 8). Inflammatory cell infiltration was indicated by black arrows. Scale bars, 50μm. (C) Representative Masson’s trichrome staining and quantitative analysis of lung sections from mice of different experimental groups (n = 6 to 8). Deposited collagen was indicated by black arrows. Scale bars, 50μm. (D) Col1a1 in mice of different experimental groups (n = 6 to 8). (E) Representative western blot images and quantitative analysis of COL1A1 in lung homogenates from mice of different experimental groups (n = 6). β-Actin was used as a loading control. (F) Lung hydroxyproline content in mice of different experimental groups (n = 6 to 7). (G) Representative images and quantitative analysis of western blots of STAT3 and SMAD2/3 phosphorylation and their total expression in lung homogenates from mice of different experimental groups. (B-G) P values were determined by one-way ANOVA. Data were expressed as mean ± SEM. *, P < 0.05; **, P < 0.01, ***, P < 0.001.

## Slide 5
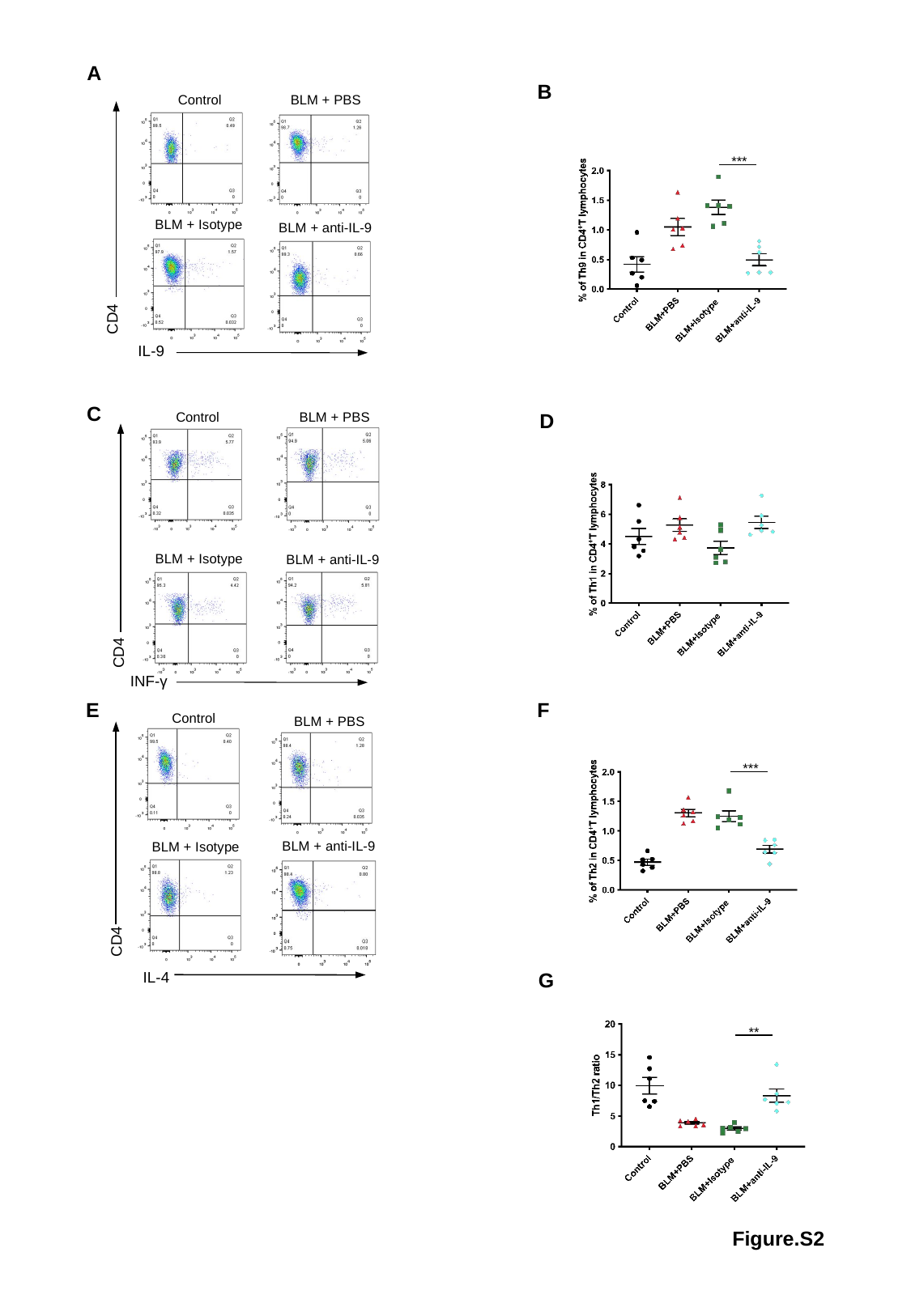

A
Control
BLM + PBS
BLM + Isotype
BLM + anti-IL-9
CD4
IL-9
B
***
C
Control
BLM + PBS
BLM + Isotype
BLM + anti-IL-9
CD4
INF-γ
D
E
Control
BLM + PBS
BLM + anti-IL-9
BLM + Isotype
CD4
IL-4
F
***
G
**
Figure.S2

## Slide 6
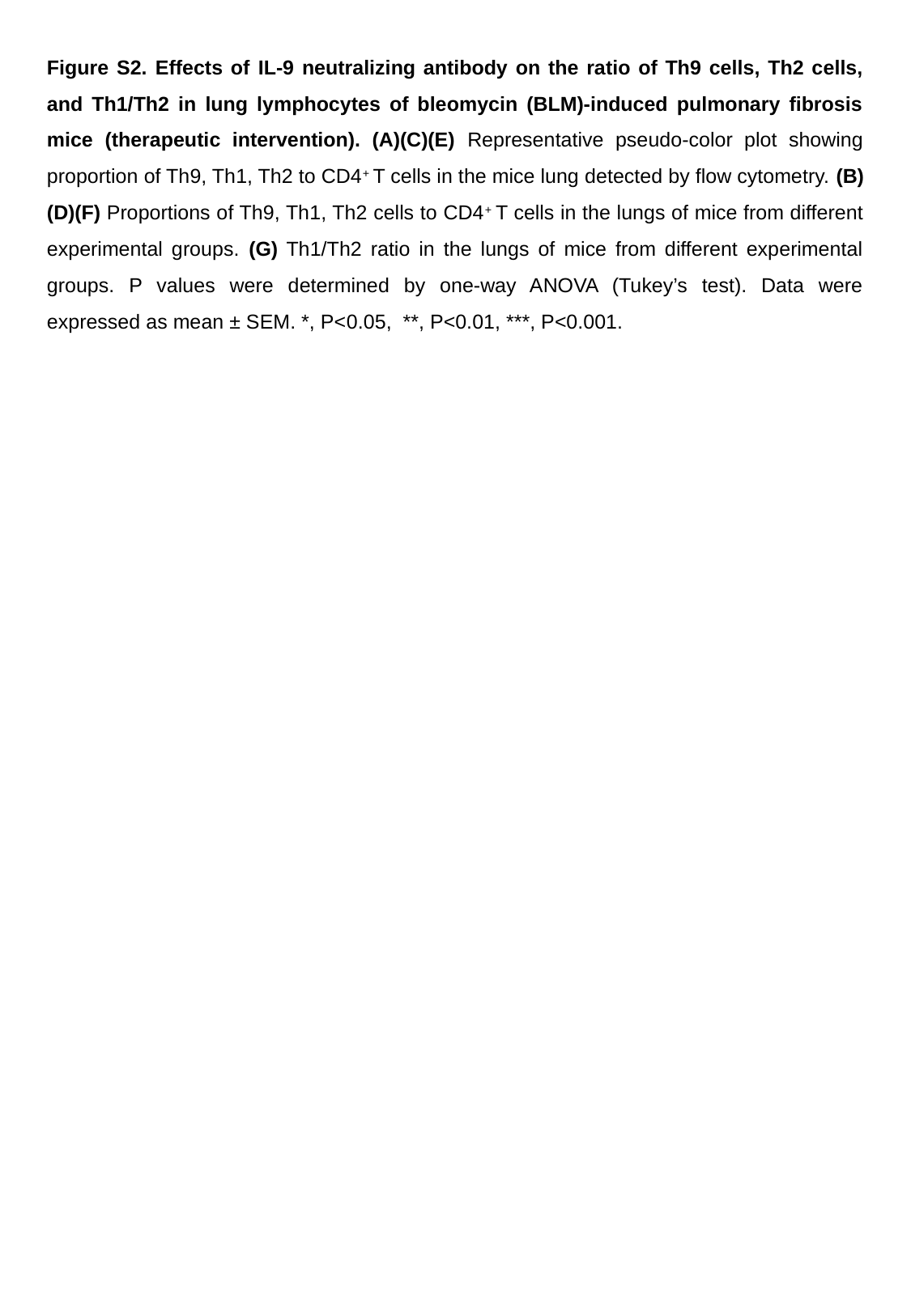

Figure S2. Effects of IL-9 neutralizing antibody on the ratio of Th9 cells, Th2 cells, and Th1/Th2 in lung lymphocytes of bleomycin (BLM)-induced pulmonary fibrosis mice (therapeutic intervention). (A)(C)(E) Representative pseudo-color plot showing proportion of Th9, Th1, Th2 to CD4+ T cells in the mice lung detected by flow cytometry. (B)(D)(F) Proportions of Th9, Th1, Th2 cells to CD4+ T cells in the lungs of mice from different experimental groups. (G) Th1/Th2 ratio in the lungs of mice from different experimental groups. P values were determined by one-way ANOVA (Tukey’s test). Data were expressed as mean ± SEM. *, P<0.05, **, P<0.01, ***, P<0.001.
